# Deconvolution of light sheet microscopy recordings

**DOI:** 10.1038/s41598-019-53875-y

**Published:** 2019-11-26

**Authors:** Klaus Becker, Saiedeh Saghafi, Marko Pende, Inna Sabdyusheva-Litschauer, Christian M. Hahn, Massih Foroughipour, Nina Jährling, Hans-Ulrich Dodt

**Affiliations:** 1 TU Wien, FKE, Dept. of Bioelectronics, Vienna, Austria; 2 Center for Brain Research, Medical University of Vienna, Vienna, Austria

**Keywords:** Imaging, Microscopy, Image processing

## Abstract

We developed a deconvolution software for light sheet microscopy that uses a theoretical point spread function, which we derived from a model of image formation in a light sheet microscope. We show that this approach provides excellent blur reduction and enhancement of fine image details for image stacks recorded with low magnification objectives of relatively high NA and high field numbers as e.g. 2x NA 0.14 FN 22, or 4x NA 0.28 FN 22. For these objectives, which are widely used in light sheet microscopy, sufficiently resolved point spread functions that are suitable for deconvolution are difficult to measure and the results obtained by common deconvolution software developed for confocal microscopy are usually poor. We demonstrate that the deconvolutions computed using our point spread function model are equivalent to those obtained using a measured point spread function for a 10x objective with NA 0.3 and for a 20x objective with NA 0.45.

## Introduction

**Figure 1:**
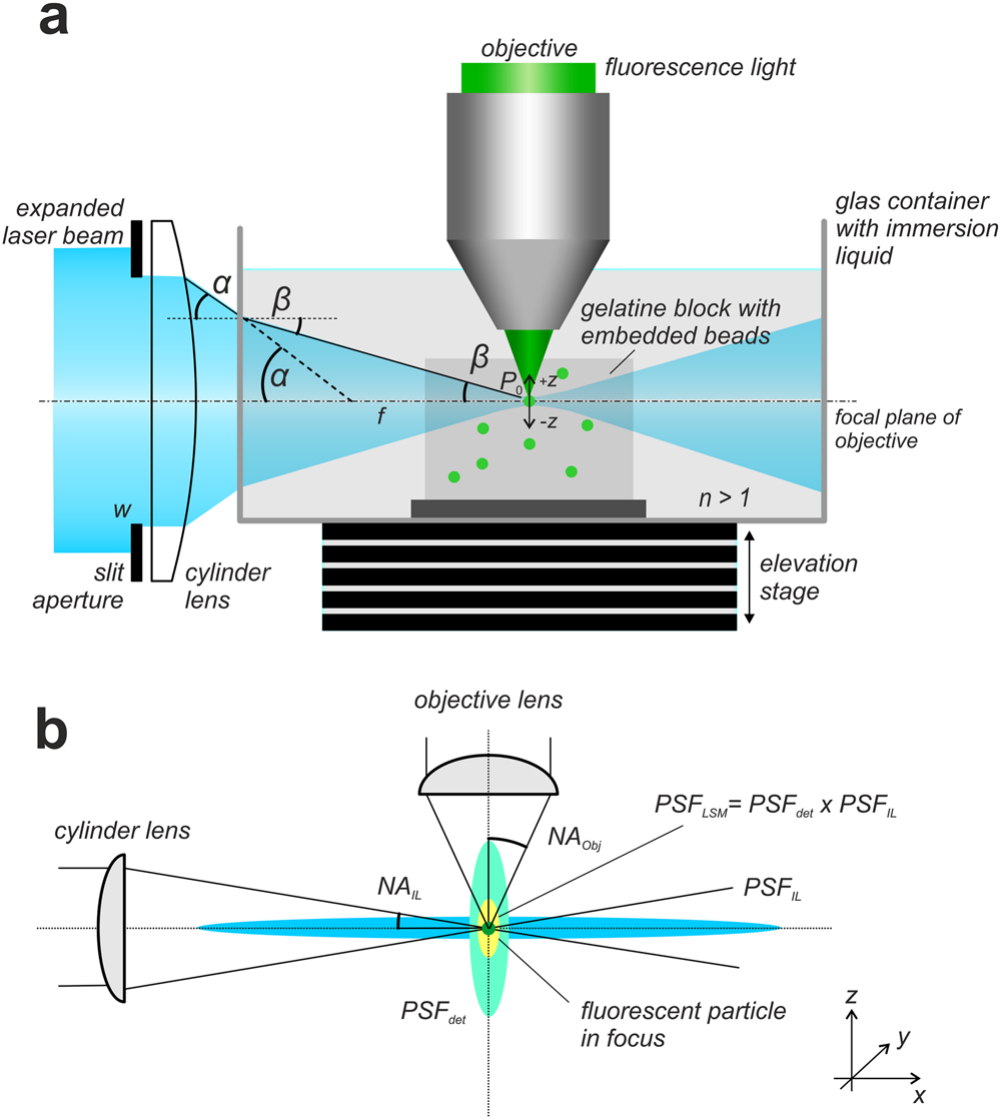
Recording of fluorescent beads. (**a**) In a light sheet microscope as described in^3^ the light sheet is generated by a sole cylinder lens and a slit aperture located directly in front of it. Incident on the slit aperture is a Gaussian distributed laser beam that has been expanded to a diameter of about 20 mm by a Galilean beam expander. Specimen and the tip of the objective are immersed in a water filled glass container. A gelatin block with embedded fluorescent particles of 200 nm diameter is placed in the focal region of the light sheet. If the system is correctly adjusted, a small number of fluorescent particles best possibly fits the focal line of the cylinder lens, as well as the focal plane of the objective. For recording, the glass container is stepwise moved vertically through the slight-sheet, while an image is recorded at each position. Since the objective (or the water-proofed protection cap mounted in front of it, respectively) is submerged in the specimen container, all optical path lengths remain constant during the recording procedure. When entering the specimen chamber, the fan angle *α* of the light sheet changes to *β*. However, due to the relation *NA*_*L*s_ = sin(*α*) = *n*sin(*β*) the numerical aperture NA_Ls_ of the light sheet remains unchanged. (**b**) A fluorescence emitting sub-resolution particle that is located in the focus of a correctly adjusted light sheet microscope is subjected to an illumination point spread function *PSF*_*IL*_ describing the spatial distribution of the excitation light close to the particle and to a detection point spread function *PSF*_*det*_ describing the near field distribution of the emitted fluorescence light collected by an objective of numerical aperture *NA*_*Obj*_. The point spread function *PSF*_*LSM*_ of the light sheet microscope can be described as the elementwise product *PSF*_*IL*_ x *PSF*_*det*_.

In recent years, light sheet microscopy became more and more common and novel variations of this technique were established^1,2^. In all these modifications, a thin sheet of laser light illuminates the specimen perpendicularly to the observation pass way of the microscope, thereby restricting fluorescence excitation to a thin layer within the specimen. This separation of illumination and observation pathway results in a pronounced optical sectioning effect^3–5^. (Fig. 1a). A major advantage of a light sheet microscope is its excellent axial resolution that, differently from a confocal microscope, can also be obtained with very low magnification objectives of relatively high NA (e.g. 2x NA 0.14) and large fields of view ranging up to more than a centimeter^3,6^.

In parallel, the still ongoing increase of memory and calculation speed of personal computers made 3D-deconvolution a well-established lab tool for debluring of microscopy data. Presently, a few commercial deconvolution software packages (e.g. Huygens, Scientific Volume Imaging Netherlands; Auto Quant, Media Cybernetics, USA) and public domain tools (e.g. Deconvolution LAB2^7^ or CLARITY^8^ are available, making 3D deconvolution accessible also to the non-specialist in computational image processing.

Standard software, using a theoretical PSF derived for wide-field or confocal microcopy provides poor results, when used for deconvolving light sheet microscopy recordings obtained with low magnification objectives. We tried out DeconvolutionLAB^7^ using two different confocal PSFs modeled with the PSF-generator^9^ plugin for ImageJ^10^ for deconvolving a light sheet microscopy stack comprising 667 optical slices obtained with a 2.5x objective (NA 0.12). We found that the results were poor or even worse compared to the original image stack (Fig. S1).

Nevertheless, the amount of available software for deconvolving light sheet microscopy data is yet low. Preibisch *et al*.^11^ and Wu *et al*.^12^ developed software that can be used for deconvolving light sheet microscopy data obtained from different directions of view (multiview combining). For the commercial products Huygens (SVI, Netherlands) and Auto Quant (Media Cybernetics, USA) add-on modules for deconvolving SPIM^5^ data exist. However, these modules have to be purchased as expensive optional extensions, rely on proprietary source code, and the price for these products is in the range of several thousand euro, making it unaffordable for many labs. To our knowledge, presently no non-commercial deconvolution tool exists, which works well for deconvolution of light sheet microscopy stacks obtained with low magnification objectives (e.g. 2x, NA0.14 or 4x NA 0.28). Here, we present an approach allowing to deconvolve such data sets in excellent quality without need of PSF measurements. We present a free software tool that can process light sheet microscopy stacks of virtually unlimited size by splitting large data sets into blocks before deconvolution and automatically stitching them afterwards. Due to its capability to use multiple CPUs and optionally GPU acceleration the program is fast, processing a stack of 1392 × 1040 × 699 pixels in less than a quarter of an hour on a NVidia P6000 graphics board (NVidia, Germany).

Light sheet microscopy combined with objectives of low magnification, but relatively high NA and large field numbers (e.g. XL Fluor 2x *NA* 0.14 FN 22, or XL Fluor 4x *NA* 0.28 FN 22, Olympus, Germany) can provide detailed images of samples of more than 1 cm size with resolutions of a few micrometer^3^. However, for these high-end objectives bridging the gap between microscopy and macro-photography, accurate point spread function (PSF) measurements that are sufficiently resolved to be applicable for deconvolution are difficult to obtain via recording of fluorescent beads. This is mainly due to the resolution limits of microscope cameras: Considering Rayleigh’s resolution criterion: *d* = 0.61*l/*NA* and further considering that according to Nyquist’s theorem the camera sampling frequency should be at least twice the highest spatial frequency resolved in the image, the required camera resolution (in megapixels) for making full use of an objective can be estimated using Rayleigh’s criterion:

1 $$\frac{2{s}_{x,y}}{V\cdot {n}_{x,y}}=\frac{0.61\lambda }{NA},\,P={n}_{x}{n}_{y}\approx 10.75\cdot {s}_{x}{s}_{y}{(\frac{NA}{V\lambda })}^{2}$$

(*P*: required number of megapixels. *NA*: numerical aperture of the objective, *λ*: emission wavelength. *V*: total magnification of the microscope. *n*_x,y_: number of pixels in *x* or *y* direction. *s*_*x,y*_: camera chip size in *x* or *y* direction).

According to Eq. (1), a camera resolution of at least 50 megapixels would be required for full use of the resolving power of e.g. a 4x objective with NA 0.28 (assumed is a typical camera chip size of 15 × 15 mm^2^, *λ* = 488 nm, no post-magnification). Unfortunately, this is much more than present microscopy cameras provide. As a consequence, recordings of sub-resolution fluorescent beads are very poorly resolved, reducing the lateral resolution of the measured PSF to a very limited number of pixels, which is not sufficient for deconvolution. Principally, undersampling could be avoided by applying high optical post magnifications (e.g. 4x or more) during bead recording. However, this approach would severely reduce the light efficiency of the microscope, yielding to very long illumination times and poor signal to noise (SNR) ratios. Further on, the effective PSF of the microscope would be altered by the post magnification itself. Light-scattering introduced by the imaging medium (usually agar or gelatin) and the refractive index mismatch between the fluorescent beads and the embedding medium are further factors, which limit the accuracy of experimental PSF measurements in practice^13^. While the former drawback can be limited by keeping the concentration of the embedding medium (usually 1–1.5% for agar or about 4% for gelatin) and the concentration of the fluorescent particles as low as possible, the latter is more difficult to prevent due to the limited set of commercially available fluorescent beads and mounting media. Cole *et al*.^14^ recommend to use the resin based embedding media ProLong Gold or Cytoseal (both available from ThermoFisher Scientific, Germany) instead of agar, which have a refractive index of 1.46 or 1.48, respectively and therefore better match the refractive index of available fluorescent microspheres. A theoretical approach describing the PSF in a complex light scattering medium has been developed by Boniface *et al*.^13^.

Our approach avoids the difficulties of PSF measurements by utilizing a computed PSF that is derived from an optical model of image formation in a light sheet microscope. This allows to tabularize the PSF on any required resolution scale. We show that this approach provides excellent deconvolutions for light sheet microscopy stacks recorded within a magnification range from 1x (e.g. 2x NA 0.14 with 0.5x post-magnification) up to 20x (e.g. 20x NA 0.45, no post-magnification) without any requirements for time consuming and error-prone PSF measurements.

## Results

### Software

The deconvolution software for light sheet microscopy that is available as additional material to this paper was written using MATLAB (The Math Works, Germany). The MATLAB script as well as a compiled stand-alone version of the software not requiring a MATLAB installation (64 bit windows only, at least 8 GB RAM required) and which also includes a simple graphical user interface is available from the Supplemental Materials.

### Validation of the PSF model

**Figure 2:**
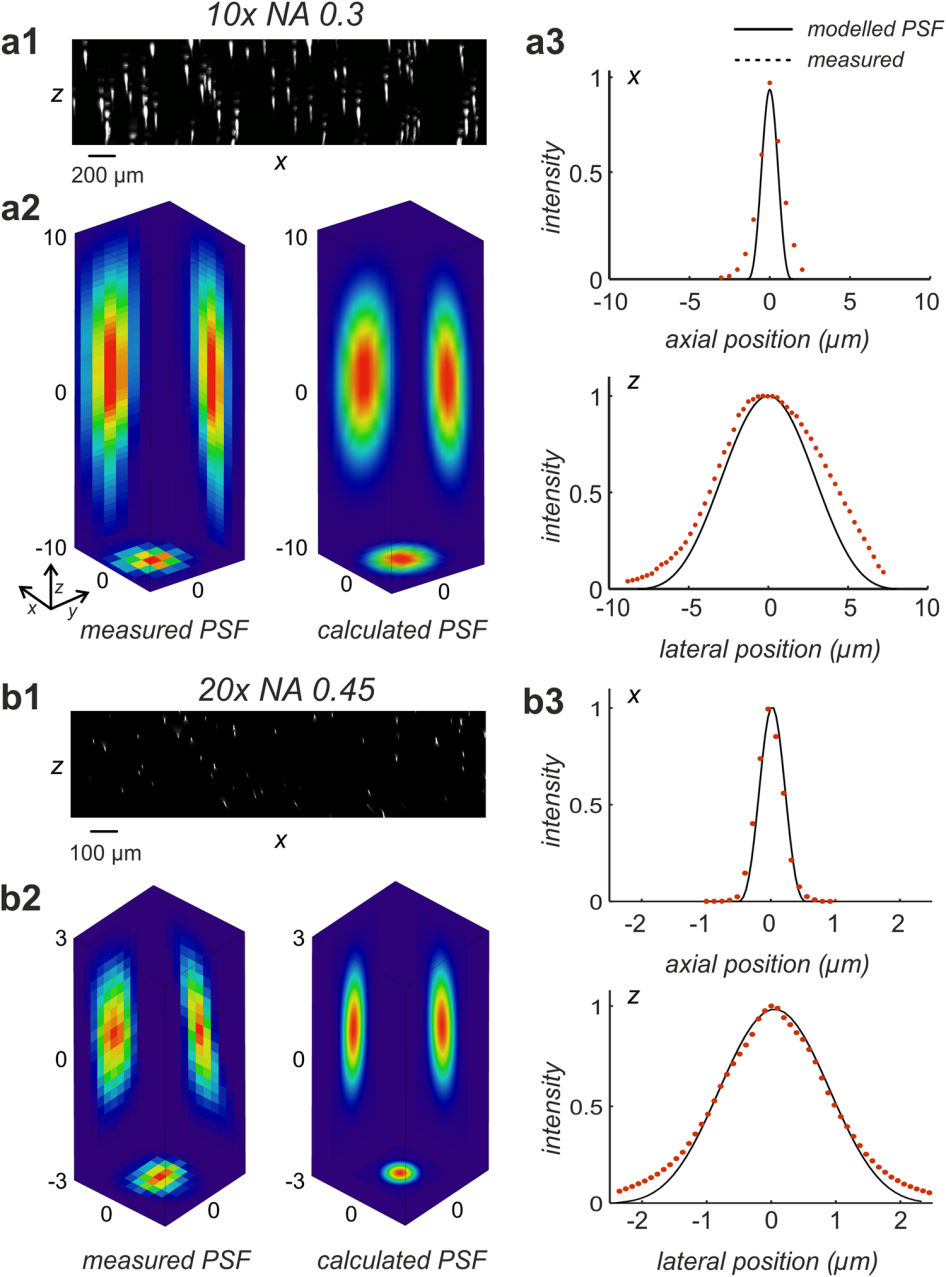
Comparison between measured and modelled PSFs of a light sheet microscope. (**a1**) 200 nm sized fluorescent beads were recorded using a 10x objective with NA = 0.3 (UPLFLN 10x, Olympus, Germany). The figure shows a maximum intensity (MIP) projection (*xz*-direction) obtained from 402 slices. (**a2**) PSF extracted by registration and averaging of 10 manually selected beads from (**a1**) (left), compared to a calculated PSF according to Eq. (15) (Model parameters: *NA* = 0.3, *λ*_*ex*_ = 488 nm, *λ*_*em*_ = 520 nm, *n* = 1.561, *f* = 80 mm, *d* = 8 mm, no damping). (**a3**) comparison of lateral (*x*, y = 0, *z* = 0) and axial (*x* = 0, *y* = 0, z) intensity profiles of the measured and the modelled PSFs depicted in (**a2**). (**b1**) 200 nm fluorescent beads were recorded using a 20x objective with NA = 0.45 (LUCPLFLN, Olympus, Germany). The panel shows a maximum intensity projection (MIP, *xz*-direction) of the 3D reconstructed fluorescent beads obtained from 500 slices. (**b2**) PSF obtained after registration and averaging of 10 manually selected beads from b1 (left) versus a calculated PSF according to Eq. (15). (Model parameters: *NA* = 0.6, *λ*_*ex*_ = 488 nm, *λ*_*em*_ = 520 nm, *n* = 1.561, *f* = 80 mm, *d* = 8 mm, no damping). (**b3**) comparison of lateral (*x*, y = 0, *z* = 0) and axial (*x* = 0, *y* = 0, z) intensity profiles of the measured and of the modelled PSFs depicted in (**b2**). As for the 10x objective visual comparison confirms a good agreement between measurements and theory.

We tested our PSF model by comparing modelled PSFs with PSFs measured using a 10x objective (NA 0.3, UPlan FLN, Olympus, Austria) and a 20x objective (NA 0.45, LUCFPLFLN, Olympus. Austria). Both objectives were custom corrected for a refractive index of 1.45. Details of the PSF measurements are described in the Supplemental Materials. For the calculated and for the measured PSFs, intensity profiles were determined and plotted into a common diagram for visual comparison. The high agreement between theory and experiment is obvious for both objectives in lateral, as well as in axial direction (Fig. 2**)**.

### Comparison of deconvolutions either obtained with a modelled or with a measured PSF

**Figure 3:**
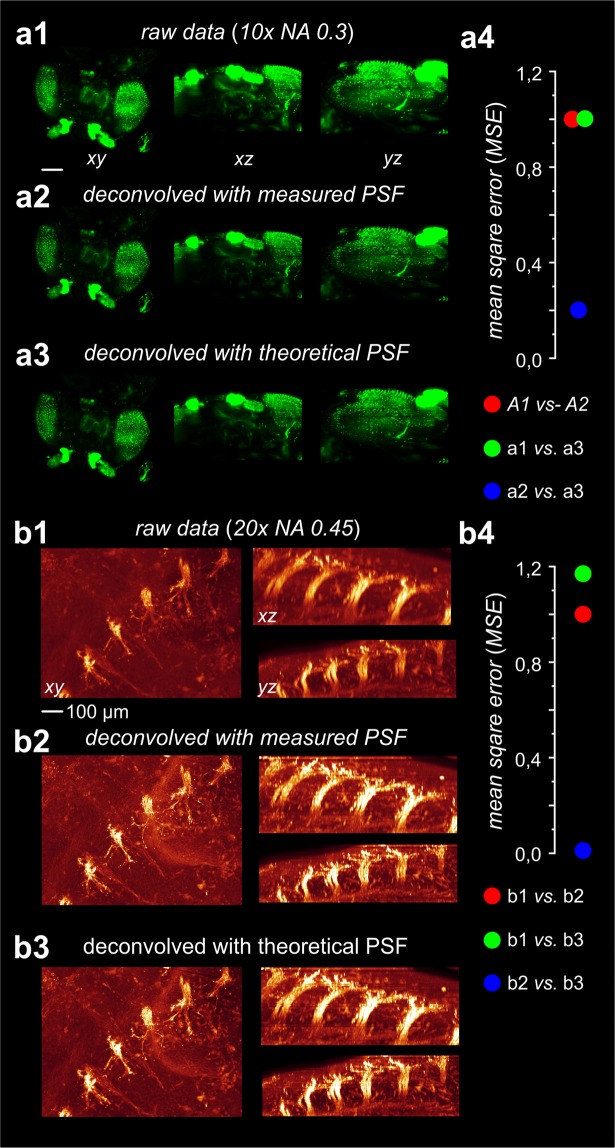
Comparison of deconvolution results either obtained using a measured PSF or a modelled PSF. (**a1**) 3D-reconstructions of a cleared fruit fly from 353 raw slices (MIP projection). Images were recorded using a 10x objective with NA = 0.3 (UPLFLN 10x, Olympus, Germany). (**a2**) Data from **a1** after deconvolution with the measured PSF depicted in **a2**. (**a3**). Data from **a1** after deconvolution with the modelled PSF depicted in **a2**. A comparison of **a2** and **a3** shows that the deconvolution results are almost identical. (**a4**) Mean standard deviations (MSD) measured between **a1** vs. **a2** (4.30 × 10^5^ = 99.8%), **a1** vs. **a3** (4.31 × 10^5^ = 100%) and **a2** vs. **a3** (9.33 × 10^4^ = 21.6%). (**b1**) Detail of a cleared mouse embryo (dorsal root ganglia) obtained from 419 raw slices (MIP projection). Images were recorded using a 20x objective with NA = 0.45 (LUCPLFLN, Olympus, Germany). (**b2**) Data from b1 after deconvolution using the measured PSF depicted in **b2**,**3**). Data from **b1** after deconvolution using the modelled PSF depicted in **b2** (right). Visual comparison of **b2** and **b3** confirms that the deconvolution results are virtually identical. (**b4**) Mean standard deviations (MSD) measured between **b1** vs. **b2** (1.37 × 10^8^ = 85.5%), **b1** vs. **b3** (1.61 × 10^8^ = 100%) and **b2** vs. **b3** (1.52 × 10^6^ = 0.95%).

By visual inspection we could not find any differences in the quality of the deconvolutions obtained with a computed PSF, or with a measured PSF for the 10x objective (Fig. 3a), as well as for the 20x objective (Fig. 3b). We did not perform such comparisons for the 2x (NA 0.14) and for the 4x (NA 0.28) objective, due to the previously addressed difficulties to perform PSF measurements of good quality with these objectives.

We quantitatively compared the raw image stacks, the image stacks obtained after deconvolving with a measured PSF and the image stacks obtained after deconvolving with a calculated PSF by means of their mean squared deviation (MSD).

2 $$MSD=\frac{1}{N}\mathop{\sum }\limits_{1}^{N}{({x}_{1,n}-{x}_{2,n})}^{2}$$

where *x*_1_ represents the voxels in the first data set and x_2_ represents the voxels in the second data set. N is the number of voxels.

As depicted in Fig. 3a4,b4 the MSD calculated between the stacks deblurred with the measured and with a computed PSF is much lower compared to the deviations from the original stacks. This strongly suggests that the results obtained using a measured PSF and those obtained using a calculated PSF are highly similar relatively to the original stack, as already evident by visual inspection. The MSD values plotted in Fig. **a4**, **b4** are normalized to the MSD calculated between the original data set and the respective data set deconvolved using a measured PSF.

### Deconvolution performance in the presence of noise

**Figure 4:**
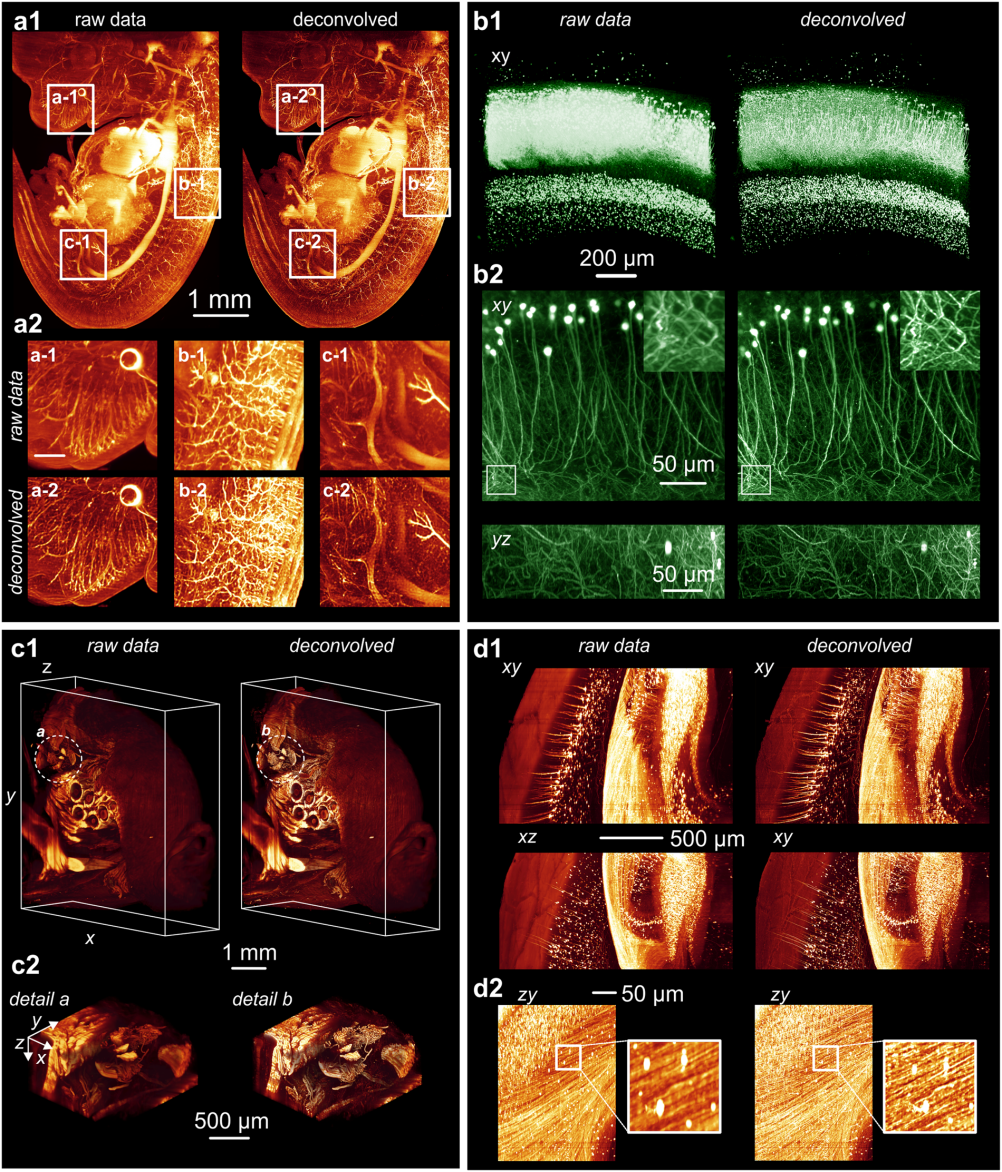
3D reconstructions from different samples prior and after deconvolution. The deconvolution approach described in this paper provides a significant increase in sharpness and in the level of details of light sheet microscopy recordings for different samples and magnifications ranging from 1x to 20x. (**a**) 3D reconstructions (MIP projection) of an E12.5 mouse embryo that was immune-stained and chemically cleared according to^32^. Nerve fibers are highlighted by NF-160 fluorescence labelling^32^. The reconstruction was obtained from 667 slices recorded using a 2.5x objective (Zeiss FLUAR 2.5x, Carl Zeiss, Germany) with an NA of 0.12 and a 0.5x post magnification. Recording was performed using a light sheet microscope equipped with a single cylindrical lens of 80 mm focal length and a 6 mm wide slit aperture as described in^3^. For imaging, a Cool Snap Cf CCD camera (Roper Scientific, Germany) with 1392 × 1040 pixel resolution was used. Illumination time: 430 ms. (**a1**) The left column shows a 3D-reconstruction obtained from the unprocessed raw data. The right side shows a reconstruction obtained from the same data set after deconvolution with a calculated PSF. (**a2**) Zoomed details from three selected regions before deconvolution (**a1**–**c1**) and after deconvolution (**a2**–**c2**). Deconvolution parameters were *NA* = 0.14, *λ*_*ex*_ = 488 nm, *λ*_*em*_ = 520 nm, *n* = 1.561, *f* = 80 mm, *d* = 8 mm, stop criterion = 0.1%, no damping. (**b**) 3D reconstructions obtained from an isolated EGFP expressing mouse hippocampus that has been chemically cleared according to^33^. (**b1**) Reconstruction obtained from 1050 slices recorded using a 5x objective (FLUAR 5x, Carl Zeiss, Germany) with a NA of 0.25 and a 0.5x post magnification (left image) and the same data set after deconvolution (right image). Recording was performed with a light sheet microscope equipped with a 80 mm cylindrical lens and a 6 mm wide slit aperture as described in^3^. For deconvolution, the data set was split into 1 × 1 × 3 equally sized blocks. Deconvolution parameters were *NA* = 0.25, *λ*_*ex*_ = 488 nm, *λ*_*em*_ = 520 nm, *n* = 1.561, *f* = 80 mm, *d* = 8 mm, stop criterion = 0.1%, no damping. Imaging was done using a Cool Snap cf camera (Roper Scientific, Germany) with 1392 × 1040 pixel resolution. Illumination time: 2000 ms. (**b2**) Reconstruction obtained from 221 slices of 1392 × 1040 pixel resolution recorded using a 20x objective (LUCPLFLN 20x, NA 0.45, Olympus, Germany) (left column) and the same data set after deconvolution (right column). Recording was performed with a light sheet microscope equipped with a 80 mm cylindrical lens and a 16 mm wide slit aperture as described in^3^. Deconvolution parameters were *NA* = 0.45, *λ*_*ex*_ = 488 nm, *λ*_*em*_ = 520 nm, *n* = 1.561, *f* = 80 mm, *d* = 16 mm, 30 iterations. Imaging was done using a Cool Snap cf (Roper Scientific, Germany) camera with 1392 × 1040 pixel resolution. Illumination time: 10000 ms. **c**) Part of the head of an entire adult mouse chemically cleared according to^34^. **c1**) Reconstruction obtained from 1297 slices recorded using a 2x objective (XLFLUOR 2x, Olympus, Germany), with an NA of 0.14 and a 0.63x post magnification (left image) and the same data set after deconvolution (right image). Recording was performed using a light sheet microscope equipped with a modified light sheet generator as described in^22^. For imaging an Andor Neo CMOS camera (Andor, Ireland) with 2560 × 2160 pixel resolution was used. Illumination time: 180 ms. Deconvolution parameters were *NA* = 0.14, *λ*_*ex*_ = 488 nm, *λ*_*em*_ = 520 nm, *n* = 1.561, *f* = 80 mm, *d* = 16 mm, 30 iterations, no damping. **c2**) Zoomed details from regions **a**-**b** marked in **c1** before deconvolution (left) and after deconvolution (right). **d**) Cortical neurons recorded in an entire mouse brain that was chemically cleared according to^35^. (**d1**) Reconstructions obtained from 777 slices recorded using a 4x objective (XLFLUOR 4x, Olympus, Germany) with an NA of 0.28 and a 2x post magnification (left column) and the same data set after deconvolution (right column). Recording was performed using a light sheet microscope with a modified light sheet generator as described in^22^. Imaging was done using an Andor Neo CMOS camera with 2560 × 2160 pixel resolution. Illumination time: 250 ms. Deconvolution parameters were *NA* = 0.28, *λ*_*ex*_ = 488 nm, *λ*_*em*_ = 520 nm, *n* = 1.561, *f* = 80 mm, *d* = 16 mm, 30 iterations, stop criterion = 0%, no damping. (**d2**) Two different zooms of **d1** before deconvolution (left) and after deconvolution (right).

**Figure 5:**
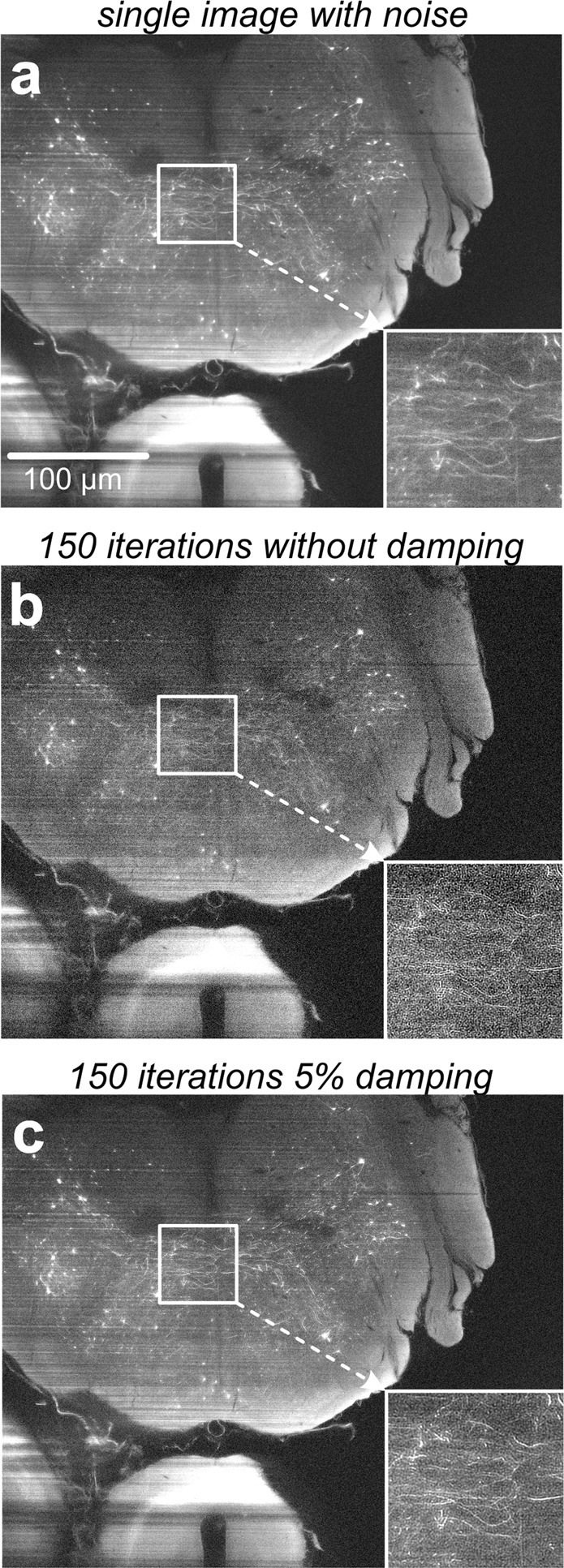
Effect of the damping parameter on noise amplification. (**a**) Single slice out of a light sheet microscopy image stack comprising 51 slices recorded from a GFP-expressing mouse brain. For demonstrating, the effect of the damping parameter, artificial computer generated Gaussian noise was added. (**b**) Deconvolution of the same data without damping causes an amplification of the noise, masking fine details of the image. The quality after deconvolution is even worse than the quality before deconvolution. (**c**) Deconvolution of the same data set using 5% damping. The amplification of noise is significantly reduced and a quality improvement compared to the raw data (**A**) is clearly recognizable now.

To improve deconvolution of image stacks with low SNR, we integrated flux preserving regularization^15^ as an option in the deconvolution algorithm. In order to analyze its effect we generated a noisy input image stack by adding computer generated Gaussian noise ($\overline{x}$ = 0, *σ*^*2*^ = 0.01) to the data set underlying Figs. 4d and 5a). After deconvolution without regularization (damping) we expectedly observed a strong amplification of the background noise masking any fine details of the image (Fig. 5b). However, repeating the deconvolution with 5% damping suppressed the noise amplification to a tolerable degree. While the level of detail in the image deconvolved without regularization is even less compared to the original image, a distinct quality improvement is obvious in the image that was deconvolved using regularization (Fig. 5c).

### Processing speed

The required deconvolution times strongly depend on the available hardware. The FFT calculations make use of multiple processor cores. On an older PC, equipped with two quad core processors (Intel Xeon E5520) running at 2.3 GHz and 48 GB RAM, deconvolution of the data stack (1392 × 1024 × 667 voxel) belonging to the mouse embryo shown in Fig. 4a required 66 minutes. According to the limited amount of RAM, the stack was automatically split into 2 × 2 × 3 equally sized blocks. On a high-end machine equipped with an 18 core processor (Intel Xeon Gold 6140) at 3.4 GHz and 256 GB RAM the same calculation took 22 minutes. Splitting of the data into blocks was not required.

We further implemented an option to perform the convolution operations on the GPU (requires a NVidia graphic board supporting at least CUDA compute level 3). With GPU processing the required deconvolution time for the mouse embryo (1392 × 1040 × 669 voxel) was reduced to about 10 min on a NVidia P6000 graphic processor board with 24 GB RAM (NVidia, Germany).

### Example deconvolutions

We carefully tested our deconvolution program with light sheet microscopy stacks obtained from different chemically cleared biological samples recorded with magnifications ranging from 1x (2x objective NA 0.14, 0.5x post magnification) up to 20x (20x objective NA 0.45, no post magnification). Details of sample preparation are provided in the Supplemental Materials. For all our test samples, we observed a distinct improvement in sharpness, as well as in the level of detail (**Fig.** **4**). Especially for the lowest magnifications (e.g. 2x NA 0.14 with 0.5x post-magnification, **Fig.** **4a**) we were not able to obtain comparably detailed deconvolutions with other deconvolution software tested.

## Discussion

In the recent years, light sheet microscopy evolved to a valuable tool for live science imaging^2^. Major strengths of this fluorescence microscopy technique are its high imaging speed, excellent signal-to-noise ratio and low levels of photo bleaching and phototoxic effects^16^. Differently to confocal microscopy, light sheet microscopy provides good optical sectioning even with objectives of very low magnifications at the threshold between microscopy and macro-photography. The novel possibility to perform optical sectioning microscopy in the very low magnification range that came up with light sheet microscopy makes it a method of choice for numerous neurobiological and developmental studies on large samples that can be chemically cleared, as e.g. whole mouse brains, embryos, or even whole transparent mice^17^. Our deconvolution software developed for light sheet microscopy provides an easily applicable way to further significantly enhance the quality of 3D-reconstructions obtained from such samples.

A light sheet microscope uses the fluorescence light that is emitted by a specimen a magnitude more efficient than a confocal microscope, since it doesn’t need a pinhole blocking the major part of the photons before reaching the camera target. This allows to use standard CCD or CMOS cameras instead of photo multipliers, as common in confocal microscopy. Therefore, the SNR of light sheet microscopy recordings generally is more than a magnitude higher compared to confocal microscopy^16^. This makes light sheet microscopy data sets ideally suited for post-processing by deconvolution, since the major drawback of deconvolution - amplification of background noise - is a much less severe problem compared to confocal microscopy. This corresponds well to our finding that for the deconvolutions presented in this paper no regularization of the Richardson-Lucy algorithm was necessary to achieve the best possible results.

The PSF of a light sheet microscope has been described as the elementwise product of an illumination PSF and a detection PSF before^5,6,18^. WU *et al*.^12^ used a related approach for image fusion of multi-view images captured by wide-field or light sheet microscopy. However, differently from us, they multiplied the detection PSF with the Gaussian illumination light sheet profile, instead of the illumination PSF^12^. We tried out this approach and found that the modeled PSFs were less similar to our measured PSFs for the 10x and the 20x objective and that the obtained deconvolutions were of less quality (Fig. S2).

Attempts to perform deconvolution with a space-variant PSF have been made by several research groups^19–21^. We also tried to consider the special variability of the light sheet, and thus of the PSF, along the propagation axis in our PSF model. Surprisingly, aside of markedly reducing the deconvolution speed and adding additional model parameters that cannot straightforwardly be determined experimentally, the observed improvement turned out to be very limited. We therefore decided to assume the light sheet as approximately uniform for better speed and operability of our deconvolution tool in practice. However, a quantification of the MSE between original and deconvolved image in six equally sized, stripe-shape regions along the light sheet propagation axis suggests a distinct dependency of the deconvolution quality on the lateral position (Fig. S3). Further progress in light sheet generation should reduce this drawback by providing more homogeneous light sheets with much longer Rayleigh ranges than possible with the used standard light sheet microscope comprising a slit aperture in combination with a single cylindrical lens^22^.

Rolling ball background substraction^23^, or contrast limited histogram equilibration^24^ (CLAHE) are alternative methods for enhancing microscopy images. Advantages of both approaches are the significantly lower requests for computation power and memory compared to deconvolution and the fact that no information about the PSF or the microscope is required. We compared both approaches with our deconvolution method (Fig. S4). We found that deconvolution provides better sharpening and image contrast compared to both techniques. However, rolling ball background subtraction may be useful for pre-processing image stacks with high background intensities and contrast limited histogram equilibration may be useful for slightly post-processing deconvolved image stacks.

We developed our deconvolution program for deconvolving light sheet microscopy recordings obtained with low magnification objectives, ranging from 1x up to about 20x, without need for time-consuming and error-prone PSF measurements. We could proof that for a 10x (NA 0.3) and for a 20x objective (NA 0.45) the deconvolutions obtained with our modelled PSF are as good as those obtained using a measured PSF (Fig. 3). For a standard light sheet microscopy setup utilizing a slit aperture of up to 16 mm width and a cylinder lens with 80 mm focal length, the minimal theoretical beam waist is about 3.7 µm (FWHM) at 488 nm wavelength. Since the axial resolution of a 20x objective (NA 0.45) at 520 nm emission wavelength is about 8–9 µm, a 20x magnification is close to the upper limit for which a reasonable optical sectioning effect can be expected for this type of setup. For higher magnification objectives (e.g. 40x or higher) with numerical apertures above 0.5 used in combination with a more focused light sheet (e.g. by using a cylindrical lens with smaller focal length), an accurate measurement of the PSF by recording of sub-resolution fluorescent beads may be preferable. However, since for these objectives the sampling rate of microscopy cameras is much better matched to the field of view, PSF measurements can be done in good quality with moderate effort. Taken together, our deconvolution tools provides a stunning enhancement of details for light sheet microscopy recordings performed with low magnification objectives.

## Methods

### PSF modeling

In a correctly adjusted light sheet microscope, a thin light sheet passes through the focal plane *P*_0_ of an objective collecting the light emitted by excited fluorochromes. During the imaging process, the positions of the light sheet and of the objective remain unchanged, while the specimen is stepwise shifted through the light sheet. If the objective (or a protection cap mounted on the tip of the objective, respectively) immerses into a liquid filled container comprising the sample, all optical path lengths remain constant during the recording procedure (Fig. 1a).

An appropriate description of the PSF of this optical arrangement has to consider the far-field limit, as well as near-field diffraction: If the input field in the input plane is E(*x*_0_*, y*_0_*, z* = 0), a 2D aperture transmitting a cylindrically symmetric field is described by *E*(*x*_0_, *y*_0_, *z* = 0 = *E*(*ρ*_0_, *z* = 0), where ${\rho }_{0}=\sqrt{{x}_{0}^{2}+{y}_{0}^{2}}$, *x*_0_ = *ρ*_0_Cos(*φ*_0_) and *y*_0_ = *ρ*_0_Sin(*φ*_0_). The diffracted field then is described by the Huygens-Fresnel diffraction integral:

3 $$E(x,y,z)\cong \frac{-i\,{e}^{ikz}{e}^{\frac{ik({x}^{2}+{y}^{2})}{2z}}}{\lambda z}\iint E({x}_{0},{y}_{0},z=0){e}^{\frac{ik({x}_{0}^{2}+{y}_{0}^{2})}{2z}}{e}^{\frac{-ik(x{x}_{0}+y{y}_{0})}{z}}d{x}_{0}d{y}_{0}.$$

Using cylindrical coordinates Eq. (3) can be rewritten as:

4 $$\begin{array}{c}E(\rho ,z)\cong \frac{-i\,{e}^{ikz}{e}^{\frac{ik({\rho }^{2})}{2z}}}{\lambda z}{\int }_{0}^{2\pi }d{\phi }_{0}\\ \mathop{\int }\limits_{Aperture}\,E({\rho }_{0},z=0){e}^{\frac{ik({\rho }_{0}^{2})}{2z}}{e}^{\frac{-ik(\rho {\rho }_{0}Cos(\phi )Cos({\phi }_{0})+\rho {\rho }_{0}Sin(\phi )Sin({\phi }_{0}))}{z}}{\rho }_{0}d{\rho }_{0}.\end{array}$$

Further considering *ρρ*_0_(Cos(*φ*)Cos(*φ*_0_) + Sin(*φ*)Sin(*φ*_0_)) = *ρρ*_0_Cos(*φ*_0_ − *φ*)

Equation (4) becomes:

5 $$E(\rho ,z)\cong \frac{-i\,{e}^{ikz}{e}^{\frac{ik({\rho }^{2})}{2z}}}{\lambda z}\mathop{\int }\limits_{Aperture}\,E({\rho }_{0},z=0){e}^{\frac{ik({\rho }_{0}^{2})}{2z}}{\rho }_{0}d{\rho }_{0}{\int }_{0}^{2\pi }{e}^{\frac{-ik\rho {\rho }_{0}Cos({\phi }_{0}-\phi )}{z}}d{\phi }_{0}.$$

Substituting ${\int }_{0}^{2\pi }{e}^{\frac{-ik\rho {\rho }_{0}Cos({\phi }_{0}-\phi )}{z}}d{\phi }_{0}$ by $2\pi {J}_{0}(\frac{k\rho {\rho }_{0}}{z})$ gives:

6 $$E(\rho ,z)\cong \frac{-2\pi i\,{e}^{ikz}{e}^{\frac{ik({\rho }^{2})}{2z}}}{\lambda z}\mathop{\int }\limits_{Aperture}\,{\rho }_{0}E({\rho }_{0},z=0){e}^{\frac{ik({\rho }_{0}^{2})}{2z}}{J}_{0}(\frac{k\rho {\rho }_{0}}{z})d{\rho }_{0}$$

which resembles the Fresnel approximation of the Kirchhoff-Fresnel diffraction. Alternatively, the field *E*(*p*, *z*) can be described in terms of the numerical aperture *NA* = *n* · *Sin*(*θ*), the refractive index (*n*), and the aperture width *a* = *z* · *Sin*(*θ*):

7 $$\begin{array}{c}E(\rho ,z)\cong \frac{-\,2\pi i\,{e}^{ikz}{e}^{\frac{ik({\rho }^{2})}{2z}}}{\lambda z}\mathop{\int }\limits_{Aperture}{\rho }_{0}E({\rho }_{0},z=0){e}^{\frac{ik({\rho }_{0}^{2})}{2z}\ast \frac{z}{z}}{J}_{0}(\frac{k\rho {\rho }_{0NA}}{na})d{\rho }_{0}\\ =\frac{-\,2\pi i\,{e}^{ikz}{e}^{\frac{ik({\rho }^{2})}{2z}}}{\lambda z}\mathop{\int }\limits_{Aperture}{\rho }_{0}E({\rho }_{0},z=0){e}^{\frac{ik({\rho }_{0}^{2})z}{2}\ast {(\frac{NA}{na})}^{2}}{J}_{0}(\frac{k\rho {\rho }_{0NA}}{na})d{\rho }_{0}.\end{array}$$

By replacing the aperture size with unity, we get:

8 $$E(\rho ,z)\cong \frac{-2\pi i\,{e}^{ikz}{e}^{\frac{ik({\rho }^{2})}{2z}}}{\lambda z}{\int }_{0}^{1}{\rho }_{0}E({\rho }_{0},z=0){e}^{\frac{ik({\rho }_{0}^{2})z}{2}\ast {(\frac{NA}{n})}^{2}}{J}_{0}(\frac{k\rho {\rho }_{0NA}}{n})d{\rho }_{0}.$$

The field intensity *H* then is:

9 $$\begin{array}{c}H=|E(\rho ,z)|{|E(\rho ,z)|}^{\ast }=\frac{4{\pi }^{2}}{{(\lambda z)}^{2}}{|{\int }_{0}^{1}{\rho }_{0}E({\rho }_{0},z=0){e}^{\frac{ik({\rho }_{0}^{2})z}{2}\ast {(\frac{NA}{n})}^{2}}{J}_{0}(\frac{k\rho {\rho }_{0NA}}{n})d{\rho }_{0}|}^{2}\\ \,\,\,\,\,=\frac{4{\pi }^{2}{E}_{0}^{2}}{{(\lambda z)}^{2}}{|{\int }_{0}^{1}{J}_{0}(\frac{kNA\rho {\rho }_{0}}{n}){e}^{ik{\Delta {\rm{\rho }}}_{0}}{\rho }_{0}d{\rho }_{0}|}^{2}\end{array}$$

where ${\Delta {\rm{\rho }}}_{0}=\frac{({\rho }_{0}^{2})z}{2}{(\frac{NA}{n})}^{2}$, $\rho =\sqrt{{x}^{2}+{y}^{2}}$ and wave number $k=\frac{2\pi }{\lambda }$.

After normalizing the intensity to unity, we get:

10 $${H}_{\det }(x,y,z)=4{|{\int }_{0}^{1}{J}_{0}(\frac{2\pi }{\lambda }\frac{N{A}_{Obj}}{n}\sqrt{{x}^{2}+{y}^{2}}{\rho }_{0}){e}^{i\frac{\pi {p}^{2}zN{A}_{Obj}^{2}}{\lambda {n}^{2}}}{\rho }_{0}d{\rho }_{0}|}^{2}\cdot $$

Equation (10) describes the detection PSF, i.e. the light intensity distribution close to a fluorescence light emitting point source that is located in the focus of an objective with the numerical aperture *NA*_*Obj*_.

The illumination PSF *H*_IL_ related to the light sheet generator system, which in its simplest form consists of a single cylinder lens and a slit aperture mounted directly in front of it, can be modelled similarly. For this, the *x* and *z* coordinates have to be flipped in Eq. (10), since the illumination pathway is turned by 90° relative to the imaging pathway (Fig. 1b). The light intensity distribution along the *y*-axis is assumed to be approximately constant (*y* = 0). This leads to an expression for the intensity distribution of the excitation light around the focal line of the light sheet generator with numerical aperture *NA*_*Ls*_:

11 $${H}_{IL}(x,y=0,z)=4{|{\int }_{0}^{1}{J}_{0}(\frac{2\pi }{\lambda }\frac{N{A}_{Ls}}{n}z{\rho }_{0}){e}^{i\frac{\pi {p}^{2}xN{A}_{Ls}^{2}}{\lambda {n}^{2}}}{\rho }_{0}d{\rho }_{0}|}^{2}.$$

In a standard light sheet microscope, where the light sheet is formed by a single cylindrical lens and an upstream slit aperture^3^ *NALs* is:

12 $$N{A}_{Ls}=\,\sin (\arctan (\frac{w}{2f}))=\frac{d}{f\sqrt{4+\frac{{d}^{2}}{{f}^{2}}}}$$

*f* denotes the focal length of the cylinder lens and *w* is the full width of the slit aperture.

The effective PSF of the light sheet microscope can be described as the elementwise product of the detection PSF *H*_*det*_ Eq. (10) and the illumination PSF *H*_*Ls*_ of the system (11)^5,6,18^ (Fig. 1b)

13 $${H}_{LSM}(x,y,z)={H}_{\det }(x,y,z)\cdot {H}_{IL}(x,y=0,z).$$

Combining Eqs (10–13) provides an expression describing the PSF of a light sheet microscope:

14 $${H}_{LSM}(x,y,z)=16{|{\int }_{0}^{1}{J}_{0}(\frac{2\pi N{A}_{Obj}\sqrt{{x}^{2}+{y}^{2}}p}{{\lambda }_{em}n}){e}^{\frac{\pi {p}^{2}zN{A}_{Obj}^{2}}{{\lambda }_{em}{n}^{2}}i}pdp|}^{2}\cdot {|{\int }_{0}^{1}{J}_{0}(\frac{2\pi dzp}{f\sqrt{4+\frac{{d}^{2}}{{f}^{2}}}{\lambda }_{ex}n}){e}^{\frac{\pi {p}^{2}x{d}^{3}}{{f}^{2}(4+\frac{{d}^{2}}{{f}^{2}}){\lambda }_{ex}{n}^{2}}i}pdp|}^{2}.$$

After rewriting using trigonometric terms, which may be preferable for implementation in a programming language that cannot handle complex exponents Eq. (14) becomes:

15 $$\begin{array}{c}{H}_{LSM}(x,y,z)={|{\int }_{0}^{1}{J}_{0}(\frac{2\pi N{A}_{Obj}\sqrt{{x}^{2}+{y}^{2}}p}{{\lambda }_{em}n})(\cos (\frac{\pi {p}^{2}zN{A}_{Obj}^{2}}{{\lambda }_{em}{n}^{2}})+i\sin (\frac{\pi {p}^{2}zN{A}_{Obj}^{2}}{{\lambda }_{em}{n}^{2}}))pdp|}^{2}\cdot \\ {|{\int }_{0}^{1}{J}_{0}(\frac{2\pi dzp}{f\sqrt{4+\frac{{d}^{2}}{{f}^{2}}{\lambda }_{ex}n}})(\cos (\frac{\pi {p}^{2}x{d}^{2}}{{f}^{2}(4+\frac{{d}^{2}}{{f}^{2}}){\lambda }_{ex}{n}^{2}})+i\sin (\frac{\pi {p}^{2}x{d}^{2}}{{f}^{2}(4+\frac{{d}^{2}}{{f}^{2}}){\lambda }_{ex}{n}^{2}}))pdp|}^{2}\end{array}$$

*x*, *y*, *z* are coordinate points, J_0_ is the Bessel function of first kind and order zero, *NA*_*Obj*_ is the numerical aperture of the objective, *λ*_*ex*_ is the excitation wavelength, *λ*_*em*_ is the emission wavelength, *f* is the focal length of the cylinder lens, and *d* is the full width of the slit aperture located in front of the cylinder lens.

**Figure 6:**
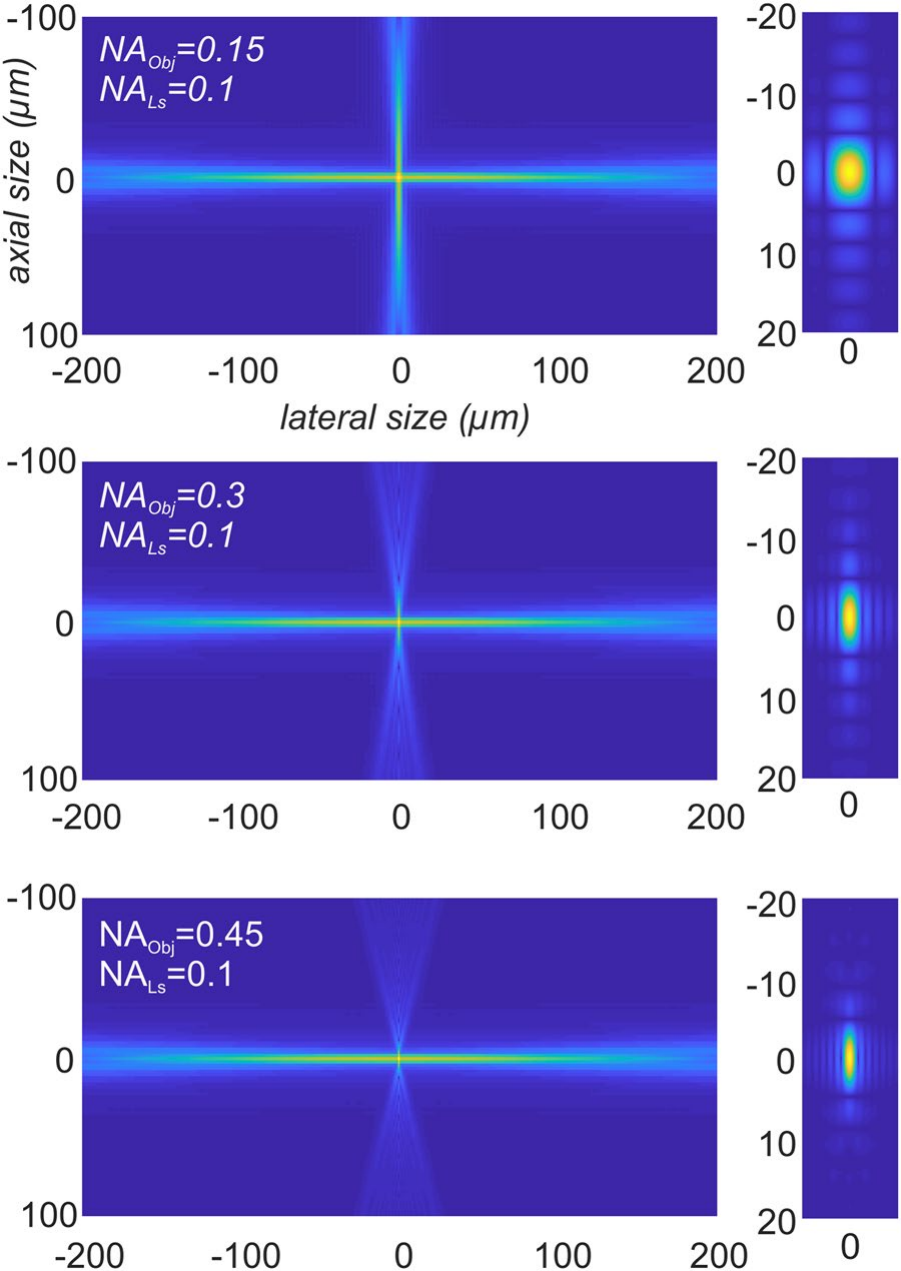
Modelling the PSF of a light sheet microscope. Simulations of *PSF*_*IL*_ and *PSF*_*det*_ of a light sheet microscope for *NA*_*LS*_ = *0.1* and *NA*_*Obj*_ = {0.15, 0.3, 0.45} (left). The according effective point spread function *PSF*_*LSM*_ is depicted on the right side. For a 2x objective with *NA*_*Obj*_ = *0.15* the effect of the detection PSF is small, since most outer parts of *PSF*_*det*_ are multiplied with values that are close to zero. However, the higher *NA*_*Obj*_ becomes, the more it effects the shape of *PSF*_*LSM*_. To enhance the visibility of the side lobes of the PSFs, the images were gamma corrected by γ = 0.4.

Since according to Eq. (15) the PSFs of the light sheet and of the objective are elementwise multiplied, the shape of the light sheet predominantly determines the PSF of a light sheet microscope, as long as its thickness is small compared to the axial size of the detection PSF^16^. Figure 6 depicts 2D-sections (*y* = 0) of PSF’s modelled according to Eq. (15) illustrating this effect.

Light sheet microscopy recordings obtained with low magnification objectives of relative high NA and large fields of view usually are severely undersampled in *xy*-direction in terms of the Nyquist rate. Hence, a PSF that is tabularized on a 3D-grid that in its lateral (*xy*) resolution matches the back projected camera pixel size *d*_*xy*_ would also be undersampled. However, according to Eq. (10) the detection PSF *H*_*det*_ does not depend on the objective magnification and, as we further pointed out, the shape of the PSF is predominantly determined by the NA of the light sheet generator and not by the objective, as long as the NA of the objective is not too high. Therefore, we can circumvent the undersampling problem addressed above by calculating the PSF on a virtual grid satisfying the Nyquist criterion, while virtually downscaling the image, accordingly. Our deconvolution software utilizes a 3D-grid with a maximal lateral spacing of ${\Delta }_{xy}=\frac{0.61\lambda }{3NA}$ for PSF calculation, which is 33% above the Nyquist rate as suggested^14^. The lateral size of the image stack is corrected accordingly. The axial spacing Δ_z_ of the calculation grid always equals the step width *d*_*z*_ for stepping the sample through the light sheet (Fig. 1a). The number of sample points along each axis (*n*_*xy*_ and *n*_*z*_, respectively) is adjusted in a way that the calculated PSF covers the range from −*FWHM* to +*FWHM* along each axis. The full width half maxima (FWHM) *FWHM*_*xy*_ and *FWHM*_*z*_ are obtained using Eq. (15) via a bisection algorithm^25^.

16 $${n}_{xy}=\frac{2FWH{M}_{xy}}{{\Delta }_{xy}}\,,\,\,{n}_{z}=\frac{2FWH{M}_{z}}{{\Delta }_{Z}}$$

*n*_*xy*_ and *n*_*z*_: number of *PSF* sample points in *xy* and *z* direction, respectively. *D*_*xy*_ and *D*_*z*_: distance between sample points in *xy* and *z* direction. *FWHM*_*xy*_: full width half maximum in *xy*-direction.

### Richardson-lucy deconvolution

Deconvolution tries to undo the blurring that is introduced by convolving the original image with the PSF of the microscope. Further, some noise, predominantly originating from the camera, sums to the image^15^.

17 $$D=O\,\otimes \,H+N=\iiint H(x-x^{\prime} ,y-y^{\prime} ,z-z^{\prime} )\,O(x^{\prime} ,y^{\prime} ,z^{\prime} )\,dx^{\prime} dy^{\prime} dz^{\prime} +N(x,y,z)$$

*D* is the recorded image stack, *O* is the restored image stack, *H* is the point spread function, and *N* is some additive noise. ⊗  symbolizes the convolution operator.

Equation (17) can be written in the frequency domain as

18 $$\Im (D)=\Im (O)\cdot \Im (H)+\Im (N),$$

where ℑ denotes the discrete 3D-Fourier transform.

In the absence of noise the convolution could be easily reverted by inverse filtering

19 $$O={\Im }^{-1}(\frac{\Im (D)}{\Im (H)})$$

where ℑ^−1^ denotes the discrete inverse Fourier transform.

However, in practice the straightforward division by ℑ(*H*) would extremely amplify the additive noise present in the image, thereby boosting high frequency components towards infinity in parts where ℑ(*H*) contains values close to zero. One of the various approaches that were developed to deal with this problem^26^ is the Richardson-Lucy (RL) deconvolution algorithm^27,28^. The RL algorithm assumes that the recorded image stack is a combination of the desired non blurred image stack that has been convolved with the PSF by the microscope optics and some additional Poisson distributed noise (17). This property makes RL deconvolution especially adequate for images recorded with CCD cameras or photomultipliers. Since these devices count numbers of photons they generally exhibit Poisson distributed noise intensity distributions^29^.

The RL algorithm tries to find an improved image stack, which, if blurred with the known PSF, best possibly matches the recorded image stack. As a first estimate, the algorithm starts with the recorded image stack. During each further iteration step *n* a correction factor is computed for each voxel of the current estimate. Then this 3D matrix of correction factors is elementwise multiplied with the current estimate to obtain the next estimate *n* + 1. For determining the correction factors, a copy of the current estimate is made and blurred by convolution with the PSF. The original stack is elementwise divided by this blurred version, yielding a new stack, which then is blurred a second time using the same PSF (in case the PSF is asymmetric it has to be flipped around its origin first). This yields the correction factors, which now can be used to obtain the estimate used for the next iteration. Equation 20 describes the basic RL algorithm.

20 $${O}^{(n+1)}={O}^{(n)}[\frac{D}{\hat{H}\otimes {O}^{(n)}}]\otimes \hat{H},\,{\hat{H}}_{(x,y,z)}={\hat{H}}_{({n}_{x}-x,{n}_{y}-y,{n}_{z}-z)}$$

*D* is the recorded image stack, *O* is the restored image stack at iteration step *n*, *H* is the point spread function, and $\hat{H}$ is the PSF flipped around its center point. ⊗ symbolizes the convolution operator.

Using the Fourier transform Eq. 20 can be written as:

21 $${O}^{(n+1)}={O}^{(n)}{\Im }^{-1}\,[\Im (\frac{D}{{\Im }^{-1}(\Im (H)\,\cdot \Im ({O}^{(n)}))})\,\cdot \Im {(H)}^{\ast }],$$

where ℑ^−1^ denotes the inverse Fourier transform, and * the complex conjugate of ℑ(*H*).

As long as the iteration converges, the difference between the current estimate and the previous estimate becomes smaller, finally approaching zero (i.e. the correction factors converge to one). In practice, either a constant number of iterations is performed (e.g. 30 iterations) or, preferably, a quality criterion *D*_*rel*_ is defined, which quantifies the difference between the current image stack and the previous image stack. We defined the normalized average squared difference *D*_*rel*_ between the output stacks obtained from two adjacent iterations as a quality criterion.

22 $${\Delta }_{{\rm{rel}}}(k)=\frac{100}{{\Delta }_{{\rm{rel}}}(1)}\cdot \frac{{\Delta }_{(k-1)}-{\Delta }_{(k)}}{{\Delta }_{(k-1)}},\,\,{\rm{with}}\,{\Delta }_{(k)}=\sqrt{\mathop{\sum }\limits_{n=1}^{N}{({O}_{n}^{(k-1)}-{O}_{n}^{(k)})}^{2}}$$

*k* is the iteration number, *D*(*k*) is the quality criterion at iteration step *k, N* is the number of voxels in the stack, and *O* is the deconvolved image stack.

Since *D*_*rel*_ is normalized by *D*_*rel*_(1) obtained from the first iteration step, *D*_*rel*_ starts with 100% and approaches 0% during the further iteration steps. In practice, the iterations are stopped when *D*(*k*) becomes smaller than a predefined stop criterion **e**.

A known limitation of the RL algorithm is its slow convergence due to oscillations and noise amplification in the presence of significant noise levels^26^. Tikhonov-Miller regularization^30^, or Total Variation Regularization are two improvements of the original RL algorithm that were developed to deal with both problems^31^. We implemented a straightforward regularization approach termed Flux-Preserving Regularization, which relies on simple spatial filtering of the intermediate data obtained in each iteration step^15^. For Flux-Preserving Regularization, a smoothed copy O*^(*n*)^ is obtained from the image stack O^(*n*)^ by convolving it with an averaging filter. When calculating the next iteration step *n* + 1 a weighted fraction γ of O*^(*n*)^ is added to obtain the next intermediate result O^(*n*+1)^.

23 $$\begin{array}{ccc}{O}^{(n+1)} & = & (1-\gamma ){O}^{(n)}[\frac{D}{H\,\,\otimes \,\,{O}^{(n)}}]\,\otimes \hat{H}+\gamma \,\Re \,\otimes \,{O}^{(n)}\\ \Re  & = & [\begin{array}{ccc}\frac{1}{26} & \frac{1}{26} & \frac{1}{26}\\ \frac{1}{26} & \frac{1}{26} & \frac{1}{26}\\ \frac{1}{26} & \frac{1}{26} & \frac{1}{26}\end{array}],[\begin{array}{ccc}\frac{1}{26} & \frac{1}{26} & \frac{1}{26}\\ \frac{1}{26} & 0 & \frac{1}{26}\\ \frac{1}{26} & \frac{1}{26} & \frac{1}{26}\end{array}],[\begin{array}{ccc}\frac{1}{26} & \frac{1}{26} & \frac{1}{26}\\ \frac{1}{26} & \frac{1}{26} & \frac{1}{26}\\ \frac{1}{26} & \frac{1}{26} & \frac{1}{26}\end{array}]\end{array}$$

γ is a weighting factor in the range between zero (no regularization) and 1. ℜ is a 3 × 3 × 3 filter kernel we used for average filtering.

## Supplementary information

Video 1

Video 2

Supplementary methods and materials

Deconvolution program

## Data Availability

The data supporting the findings of this study are available from the corresponding author upon reasonable request.

## Code availability

The source code of the program used for obtaining the deconvolutions presented in this paper is available in the supplementary software. For windows (64-bit at least 8 GB RAM) there is also a compiled version available, which does not require MATLAB.
