# Supplementary material for: Deconvolution of light sheet microscopy recordings
Source: Sci Rep. 2019 Nov 26;9:17625. doi: 10.1038/s41598-019-53875-y (PMC6879637; doi:10.1038/s41598-019-53875-y)
Supplement: Supplementary file 3 — Supplementary methods and materials [file 41598_2019_53875_MOESM3_ESM.pdf]

Deconvolution of light sheet microscopy recordings

Klaus Becker[1,2], Saiedeh Saghafi[1], Marko Pende[1,2], Inna Sabdyusheva-Litschauer[1,2], Christian M. Hahn[2], Massih Foroughipour[1,2], Nina Jährling[1,2], Hans-Ulrich Dodt[1,2].

[1]TU Wien, FKE, Dept. of Bioelectronics, Vienna, Austria.

[2]Center for Brain Research, Medical University of Vienna, Vienna, Austria.

Additional methods

Software. We used MATLAB (Math Works, Germany) to implement our deconvolution tool and compiled it into a stand-alone Windows application using the MATLAB compiler. The source code, as well as the compiled version (requires 64 bit Windows version and at least 8 GB of RAM) can be downloaded from (http://www/xxx). Running the program requires the prior installation of the MATLAB Runtime libraries, which can be obtained free of charge e.g. from https://www.mathworks.com/products/compiler/mcr. The program ("LsDeconv.exe") can either be started from a windows console window (see the attached README.TXT file for command line parameters), or launched using a simple graphical user interface (LsDeconvGUI.exe). The deconvolution tool splits image stacks that are too big to fit into the RAM automatically into separate blocks so that even very large data sets can be processed (up to ~30 GB tested). As a single limitation, at least one entire *z*-layer must fit into the RAM after splitting the data into blocks, i.e. if the deconvolution is e.g. split into 3 x 4 x 5 (*x y z*) blocks, at least 3 x 4 = 12 data blocks must fit into the RAM for final stitching. For a computer equipped with 32 GB RAM this limits the size of a single camera image to approximately 8000 pixel x 8000 pixel. There is no limitation for the number of images in *z*-direction.

PSF measurements. For PSF measurements, fluorescent beads with 200 nm diameter (FluoSpheres™ Carboxylate-Modified Microspheres, yellow-green fluorescent (505/515), 2% solids, order no. F8811 Thermo Fisher Scientific, Austria) were embedded in gelatin. We found that gelatin from pork skin (Sigma-Aldrich, Austria, order no. G1890.~4% in water) exhibits higher

transparency and less stray light generation than the more common embedding media agar or agarose. For preparing the gelatin blocks, the original vial containing 10 ml bead emulsion was carefully vortexed and a 1:100 pre-dilution in water was prepared. 2 g gelatin were dissolved in 50 ml boiling water. After cooling down to about 60°C, 2µl of the pre-diluted bead suspension were added and the mixture was carefully stirred on a magnetic stirrer. Histology forms of 7 x 7 x 5 mm ^{3} size, made from polystyrene (Plano GmbH, Germany, order-no. 2747-1) were filled with the mixture and stored in the fridge for at least 30 min to let the gelatin polymerize. The forms were wrapped into a piece of wet tissue to prevent them from drying and stored at 4°C. Before use, a gelatin block was carefully taken out of its form and glued on a metal plate of 15 x 15 x 2 mm ^{3} with a small drop of methacrylate glue to prevent it from floating in the specimen container.

The metal plate with the mounted gelatin block was submerged in the water-filled specimen chamber of the LSM and recorded using a 10x Objective (NA 0.3, UPlan FLN, Olympus, Austria) and a 20x objective (NA 0.45, LUCFPLFLN, Olympus, Austria), both without post-magnification. Light sheet generation was done using a plano-convex cylinder lens of 80 mm focal length combined with a slit aperture of 8 mm diameter mounted in front of it [1] (**Figure 1**). For fluorescence excitation a 200 mW sapphire laser (Coherent, Germany) with 488 nm wavelength was used. Images were captured using an Andor Neo scientific grade CMOS camera with 2560 x 2160 pixel resolution (Andor Technologies, Ireland). From the recorded stacks PSFs were extracted from 10 manually selected beads. Registration and averaging of beads was semi-automatically performed by the PSF-extraction module of the Amira 6.7 Visualization Software (ThermoFisher, Germany)

Sample preparation. All LSM recordings presented in this paper were obtained from chemically cleared mice and fruit flies. Animal care and euthanasia was done in accordance with the Austrian animal protection law. According to §2 of the Austrian animal experiments act, special approval by an ethics committee or an approval number was not required, since no experimental procedures were performed on living animals. The GFP expressing mice used in this study were breaded at the Anna Spiegel Animal Housing facility of the Medical University Vienna, which is supervised by the ethics committee of the Medical University of Vienna. The maintenance of the GFP mouse line was approved by the Austrian ministry of Science (BMWFW, Bundesminsterium für Wissenschaft, Forschung und Wirtschaft) at 12.01.2017 by writing (Geschäftszahl (GZ): BMWFW-66.009/0003-WF7V/3b/2017).

The GFP expressing *drosophila melanogaster* depicted in **Fig. 3a** was prepared and chemically cleared as described in [2]. The mouse embryo shown in **Fig. 3b** and **Fig. 4a** was prepared, immune-stained and rendered transparent as described in [3]. The GFP-expressing mouse hippocampus presented in **Fig. 4b** was prepared and chemically cleared as described in [4]. The whole mouse from which the head is depicted in **Fig. 4c** was prepared and entirely chemically cleared as described in [5]**.** The whole brain of a GFP expressing mouse used for **Fig. 4D** was prepared and chemically cleared according to [6].

Additional Results

Comparison of our program with DeconvolutionLAB using the PSF-generator plugin.

We compared the performance of our deconvolution program with the DeconvolutionLAB [7] deconvolution tool available as a plugin for ImageJ [8] (http://bigwww.epfl.ch/deconvolution/). The PSFs used for deconvolution were obtained using the PSF-generator [9] plugin available for ImageJ (http://bigwww.epfl.ch/algorithms /psfgenerator/). With our approach, a distinct improvement in image quality was visible (as already evident by **Fig. 4A**). However, the results obtained with DeconvolutionLab utilizing two different PSF models developed for confocal and wide-field microscopy (Gibson-Lanni [10] and Born and Wolf [9]) calculated with the PSF-generator plugin were unsatisfactory. (**Figure S1**). This strongly suggest that for deconvolving light sheet microscope recordings with low magnification objectives existing programs designed for confocal or wide-field microscopy are inappropriate. Noticeably, the quality of **Fig. S1 c1, c2, and d2** is even worse compared to the original image. We further found that our program (compiled MATLAB code) runs significantly faster than DeconvolutionLab (**Figure 1**).

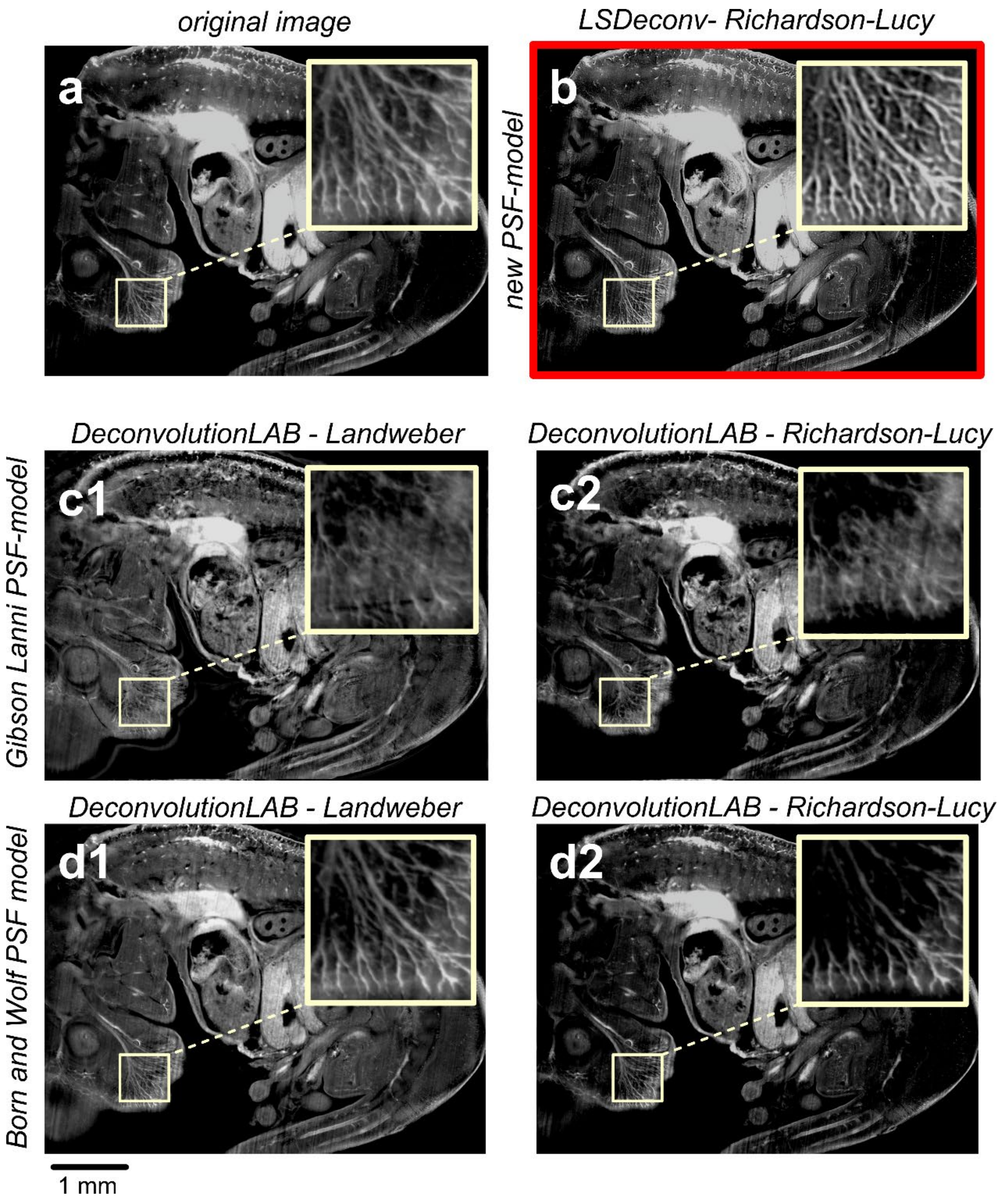

Figure S1: Comparison of our deconvolution program with DeconvolutionLAB applying two different PSFs obtained with the ImageJ PSF-generator plugin. a) Singleoptical slice from the stack used for generating Figure 4A (Zeiss Fluar 2.5x, NA 0.12, Carl Zeiss, Germany). **b**) Same optical slice after deconvolution with our deconvolution program and a modeled PSF according to eq. 15 in the main article. (10 iteratations, no damping). **c1-c4**: Same image as in a and b deconvolved with DeconvolutionLAB [7] using the Landweber [11] algorithm (C1, D1), or the RL algorithm (**c2**, **d2**), respectively. The PSFs were modeled with the ImageJ PSF-generator [9] using the Gibson- Lanny model (**c1**, **c2**) or the Born and Wolf model (**d1**, **d2**). In all cases the number of iterations was fixed to ten rounds and no damping or pre-processing was used. Required

deconvolution times: (B): 6:04 minutes without GPU support. **c1**: 18 minutes, **c2**: 03:11 hours. **d1**: 17:40 minutes, **d2**: 03:17 hours (18 core Intel Xeon Gold 6140 processor at 3.4 GHz, 256 GB RAM) .

Comparison of our PSF model with an alternative PSF model derived for light sheet microscopy.

For performing multi-view combining of wide-field and light sheet microscopy stacks Wu et al., developed a PSF model of a light sheet microscope, where a modelled wide-field PSF is elementwise multiplied with the Gaussian light sheet intensity profile [12]. We compared deconvolutions obtained with this alternative PSF modeling approach with deconvolutions obtained using our model. In accordance with WU et al., we modeled the wide-field PSF with the PSF Generator plugin developed by Kirshner et al. [9] (http://bigwww.epfl.ch/algorithms/psfgenerator/) using the "Born and Wolf" model. The subsequent multiplication with a Gaussian light sheet profile with FWHM = 3662 nm (same as used for deconvolutions with our software) was performed using MATLAB (The Mathworks, Germany).

Using this PSF, Richardson-Lucy deconvolution of two data sets that have been deconvolved with our software before, was done via the Deconvolution Lab [7] plugin available for ImageJ (http://bigwww.epfl.ch/deconvolution/) using the RL algorithm. The number of iterations was chosen identically in all cases and no damping or further processing was applied. As demonstrated by **Fig. S2**, the modeled PSFs were less similar to our measured PSFs for the 10x as well as for the 20x objective and the deconvolved images were less sharp.

Modelling the PSF of the light sheet microscope according to model B (i.e. by straightforwardly multiplying the detection PSF with the diameter of the beam waist of the Gaussian illumination light sheet profile) underestimates the size of the PSF (especially in its axial

direction) since no information about the broadening of the light sheet left and right from the exact focus point is considered in this model. Contrarily, our model (model A) considers this effect by considering the numerical aperture of the light sheet generator NA_{LS} (eq. 11), instead of assuming a constant thickness *d* of the illumination light sheet as in model B. Therefore, the axial size of the Illumination PSF H_{IL} is always somewhat bigger than the width of the light sheet at its focus. Since in our model (model A) the detection PSF H_{det} (eq. 10) is elementwise multiplied with the illumination PSF H_{IL} (eq. 11), the size of the effective PSF H_{LSM} (eq. 13) is bigger compared to model B.

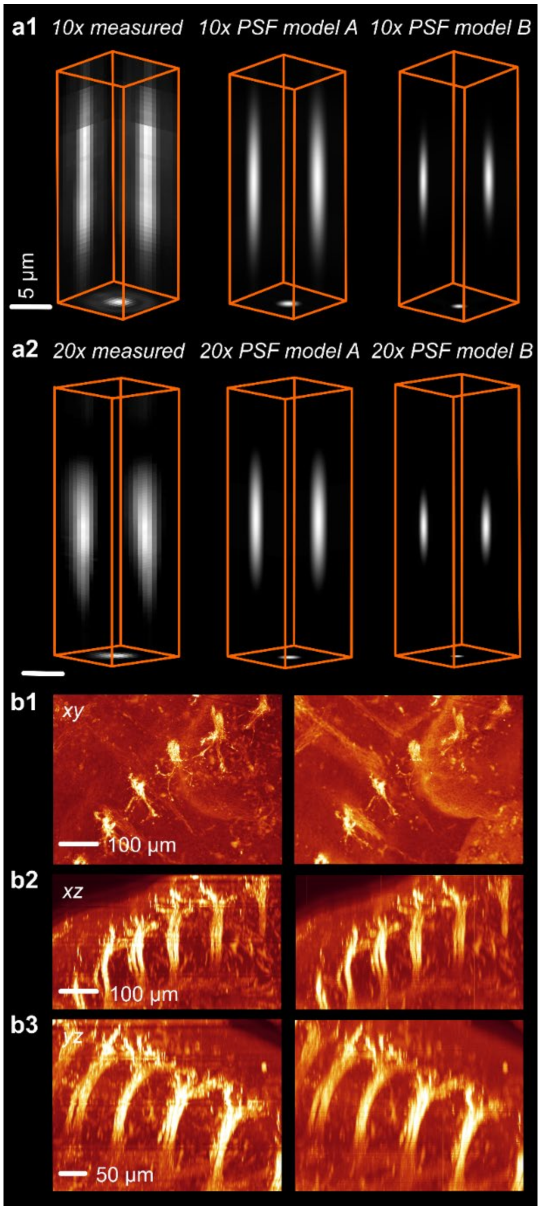

Figure S2: Comparison of two different PSF models for a light sheet microscope. a1) Measured PSF for a 10x Objective with NA 0.3 (UPLFLN 10x, Olympus, Germany), and the according PSF modelled according to eq. 15 (model A), and the respective PSF modeled according to WU et al. [12] by elementwise multiplying a wide-field PSF with a Gaussian light sheet intensity profile (model B). For models A and B a FWHM of the Gaussian light sheet of 3.662 µm was assumed. Length of scale bar: 5 µm. **a2)**: Same as a 1, however for the 20x objective with NA 0.45 (LUCPLFLN, Olympus, Germany). Length of scale bar 2 µm. **b1)-b3)**: Details of a chemically cleared mouse embryo (dorsal root ganglia) reconstructed from 419 slices. Left side: Deconvolution obtained using PSF model 1 and our software. Right side: Deconvolution obtained using PSF model B and the ImageJ [8] plugin DeconvolutionLab2 [7]. **b1**) xy-view. Length of scale bar 100 µm. **b2**): xz-view. Length of scale bar 100 µm. B3: yz-view. length of scale bar 50 µm.

Change of deconvolution efficacy along the light sheet propagation axis

We determined the mean squared error (MSE) between original and deconvolved image stack within six equally sized stripes along the light sheet propagation axis (**Figure S3**). We found that after 30 iterations of RL-deconvolution, the mean squared differences are highest in the center position, corresponds to the location of the beam waist of the illumination light sheet (**Figure 1a**). The curve reflects the broadening of the light sheet with increasing distances of the focus. The light sheet was generated using a single cylindrical lens of 80 mm focal length and a 6 mm wide slit aperture.

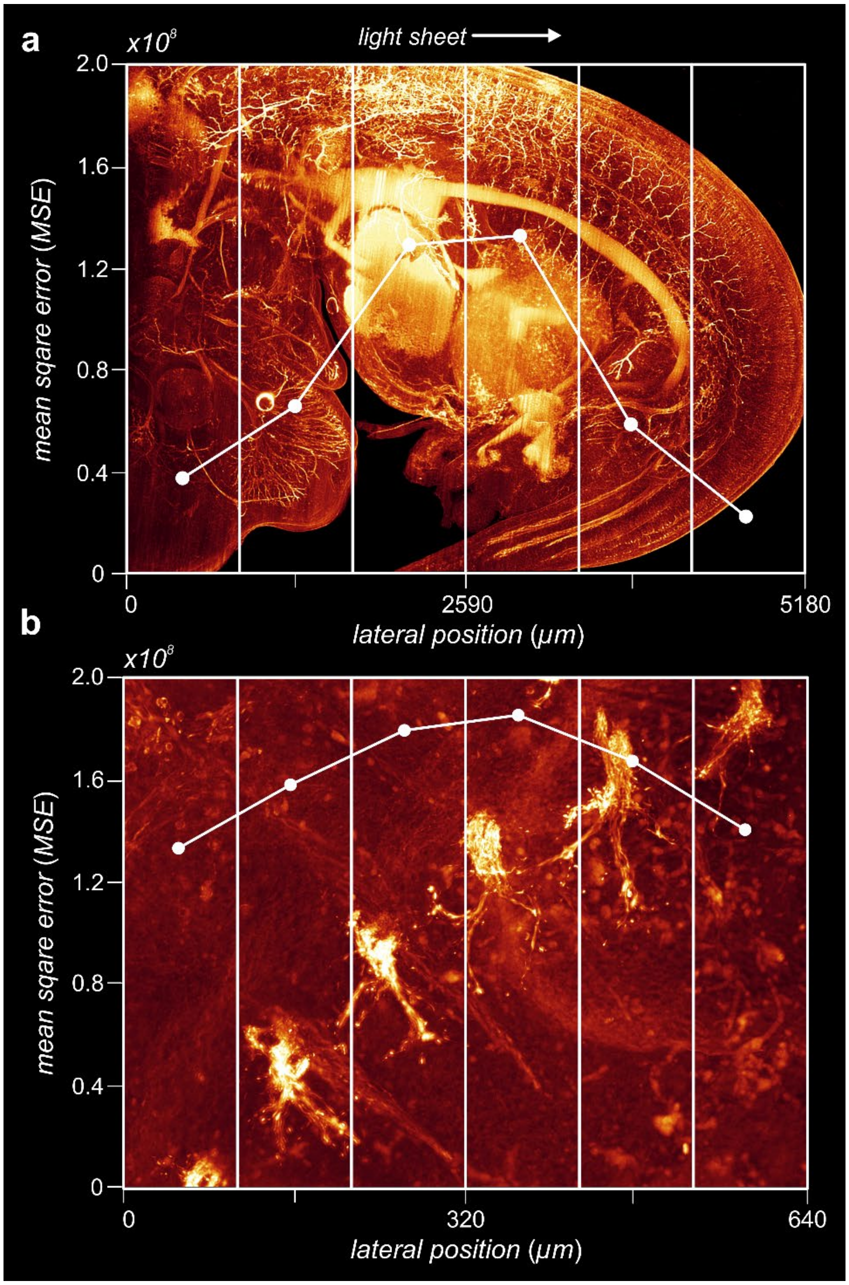

Figure S3: Variation of deconvolution efficacy along the light sheet propagation axis. A) 3D reconstruction of the same data set as depicted in **Figure S2** (2x, NA 0.14). The MSE was quantified between original and deconvolved image stack and plotted along five vertical stripes along the light sheet propagation axis. B) Same as in **a**, but for the data set shown in **Figure 1b** (20x objective, NA 0.45).

Comparison of deconvolution results with other image enhancement methods

We compared the results obtained by our deconvolution algorithm using a computed PSF with two other image enhancement techniques frequently used in computational post-processing of microscopy data: a) rolling ball background subtraction [13] and b) contrast limited histogram equilibration (CLAHE) [14] (**Figure S4**). Compared to deconvolution both techniques have the advantage that they are computationally less expensive and therefore can be performed almost in real time.

Alternatively to deconvolution we processed the image stack of the mouse embryo depicted in **Fig. 4** by the rolling ball background filter [15] implemented in ImageJ [8] and by the CLAHE algorithm implemented in Matlab R2018b (Mathworks, Germany). The radius of the ball used for rolling ball background subtraction was set to 50 pixel, the parameters chosen for CLAHE were NumTiles = [16, 16], ClipLimit = 0.001, NBins = 1000, range = 'original' and distribution = 'uniform'.

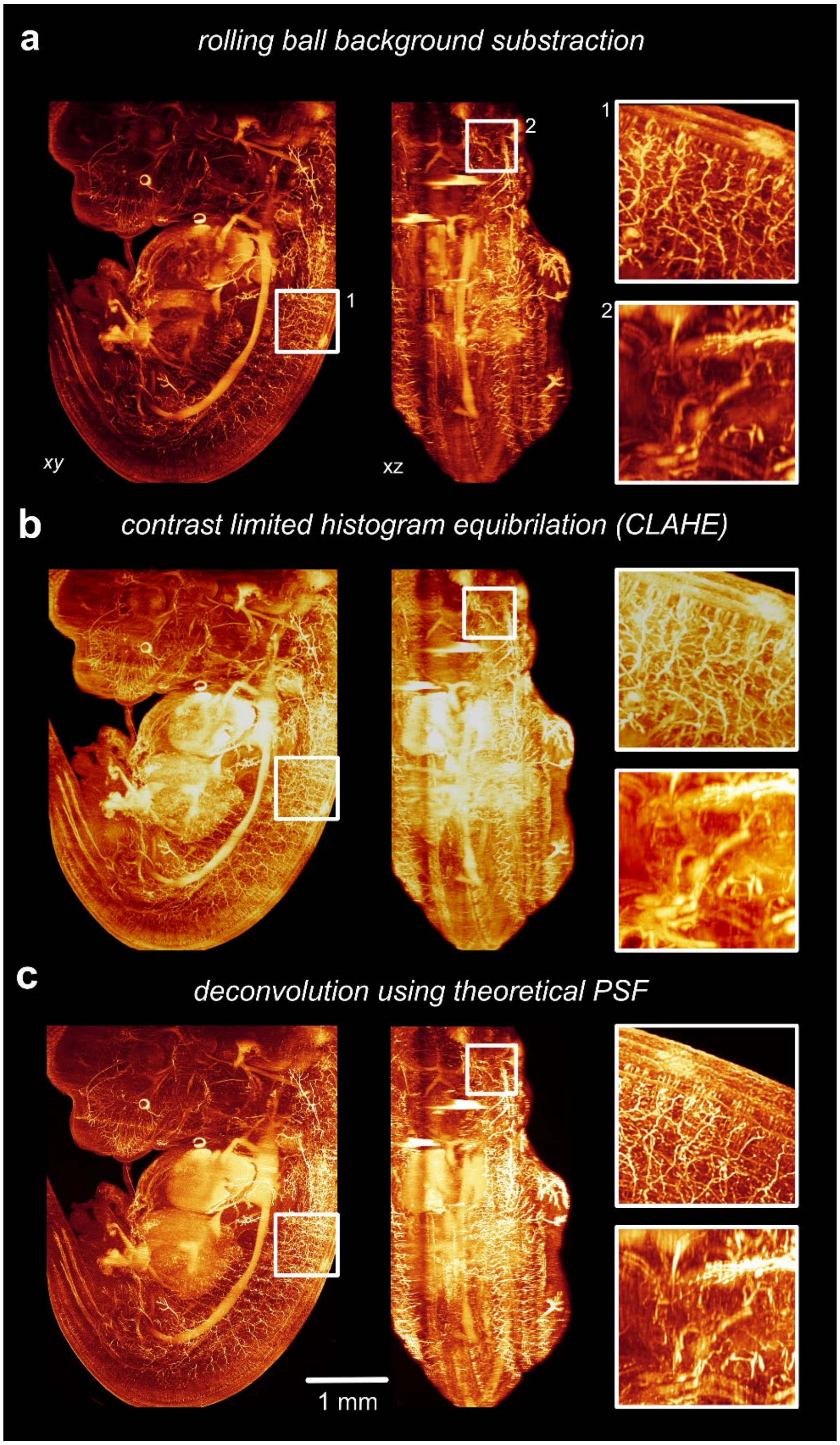

Figure S4: Comparison of the deconvolution algorithm with other non-deconvolution based image enhancement algorithms. The images show 3D reconstructions of an immune-stained, chemically cleared E12.5 mouse embryo. The reconstructions were obtained from 667 slices recorded using a 2.5x objective with a NA of 0.12 (Zeiss Fluar 2.5x, Carl Zeiss, Germany). **a**) Rolling ball background substraction. **b**) contrast limited histogram equilibration (CLAHE). **c**)

Deconvolution obtained using a modelled PSF. Although, **a** and **b** provide an obvious improvement in image sharpness and detail (compare **Fig. 4a** in the main text), superior results are obtained by deconvolution (**c**).

Supplemental videos

Video S1

Same mouse embryo as depicted in **Fig. 4a**. The left side of the video shows a 3D-reconstruction obtained from the non-deconvolved data set. The right side shows the same data set after deconvolution. For final contrast enhancement both data sets were subjected to contrast-limited histogram equilibration using identical sets of parameters (CLAHE). Nerve fibers are highlighted by NF-160 fluorescence labelling. The 3D-reconstructions were obtained from 667 slices recorded using a 2.5x objective (Zeiss FLUAR 2.5x, Carl Zeiss, Germany) with an NA of 0.12 and a 0.5x post magnification.

Video S2

Whole EGFP expressing mouse brain that has been chemically cleared with the same technique as in **Fig. 4d**. The left side of the video shows a 3D-reconstruction obtained from the non-deconvolved data set. The right side shows the same data set after deconvolution. For final contrast enhancement both data sets were subjected to contrast-limited histogram equilibration using identical sets of parameters (CLAHE). Reconstructions were obtained from 2520 slices with 2560 x 2160 pixel resolution . For imaging a 2x objective (XLFLUOR 2x, Olympus, Germany) with an NA of 0.14 and a 0.5x post magnification was used.

Supplemental references:

1. Dodt, H.-U. H.-U. *et al.* Ultramicroscopy: three-dimensional visualization of neuronal networks in the whole mouse brain. *Nat. Methods* **4**, 331–6 (2007).
2. Pende, M. *et al.* High-resolution ultramicroscopy of the developing and adult nervous system in optically cleared Drosophila melanogaster. *Nat. Commun.* 1–12 (2018). doi:10.1038/s41467-018-07192-z
3. Becker, K., Jährling, N., Kramer, E. R., Schnorrer, F. & Dodt, H. U. *Ultramicroscopy: 3D reconstruction of large microscopical specimens*. *Journal of Biophotonics* **1**, 36–42 (2008).
4. Ertürk, A. *et al.* Three-dimensional imaging of solvent-cleared organs using 3DISCO. *Nat. Protoc.* **7**, 1983–1995 (2012).
5. Perrin, D. *et al.* Whole-Body Imaging with Single-Cell Resolution by Tissue Decolorization. *Cell* **159**, 911–924 (2014).
6. Hahn, C. *et al.* High-resolution imaging of fluorescent whole mouse brains using stabilised organic media (sDISCO). *J. Biophotonics* e201800368 (2019). doi:10.1002/jbio.201800368
7. Sage, D. *et al.* DeconvolutionLab2: An open-source software for deconvolution microscopy. *Methods* **115**, 28–41 (2017).
8. Abràmoff, M. D., Hospitals, I., Magalhães, P. J. & Abràmoff, M. Image Processing with ImageJ. *Biophotonics Int.* **11**, 36–42 (2007).
9. Kirshner, H., Sage, D. & Unser, M. 3D PSF models for fluorescence microscopy in ImageJ. *… Appl. Fluoresc. …* **1**, 2010 (2011).
10. Gibson, S. F. & Lanni, F. Experimental test of an analytical model of aberration in an oil-immersion objective lens used in three-dimensional light microscopy. *J. Opt. Soc. Am. A* **9**, 154 (1992).
11. Landweber, L. An Iteration Formula for Fredholm Integral Equations of the First Kind Author (s): L . Landweber Source : American Journal of Mathematics , Vol . 73 , No . 3 (Jul ., 1951), pp . 615-624 Published by : The Johns Hopkins University Press Stable URL : ht. **73**, 615–624 (1951).
12. Wu, Y. *et al.* Simultaneous multiview capture and fusion improves spatial resolution in wide-field and light-sheet microscopy. *Optica* **3**, 897 (2016).
13. Sternberg, S. R. Biomedical Image Processing. *Computer (Long. Beach. Calif).* **16**, 22–34 (1983).
14. Pizer, S. M. *et al.* Adaptive Histogram Equalization and Its Variations. *Computer Vision Graphics and Image Processing* **39**, 355–368 (1987).
15. Schneider, C. A., Rasband, W. S. & Eliceiri, K. W. NIH Image to ImageJ: 25 years of image analysis. *Nat. Methods* **9**, 671 (2012).
