# Supplementary material for: Deconvolution of light sheet microscopy recordings
Source: Sci Rep. 2019 Nov 26;9:17625. doi: 10.1038/s41598-019-53875-y (PMC6879637; doi:10.1038/s41598-019-53875-y)
Supplement: Supplementary file 4 — Deconvolution program [file 41598_2019_53875_MOESM4_ESM.zip › LsDeconv/SourceCode.pdf]

```
% Program for Deconvolution of Light Sheet Microscopy Stacks.
% Copyright TU-Wien 2019, written using MATLAB 2018b by Klaus Becker
% (klaus.becker@tuwien.ac.at)
% LsDeconv is free software: you can redistribute it and/or modify it under the terms of the
% GNU General Public License as published by the Free Software Foundation, either version 3 of the
% License, or at your option) any later version.
% LsDeconv is distributed in the hope that it will be useful, but WITHOUT ANY WARRANTY; without
% even the implied warranty of MERCHANTABILITY or FITNESS FOR A PARTICULAR PURPOSE.  See the GNU
% General Public License for more details. You should have received a copy of the GNU General Public
% License along with this file. If not, see <http://www.gnu.org/licenses/>.

function [] = LsDeconv(varargin)
    try
        disp(' ');
        disp('LsDeconv: Deconvolution tool for Light Sheet Microscopy.');
        disp('(c) TU Wien, 2019. This program was written in MATLAB V2018b by klaus.becker@tuwien.ac.at');
        disp(' ');

        if nargin ~= 15
            showinfo();
            if isdeployed
                exit(1);
            end
            return
        end

        %read command line parameters
        inpath = varargin{1};
        dxy = varargin{2}; dz = varargin{3};
        numit = varargin{4}; NA = varargin{5}; rf = varargin{6};
        lambda_ex = varargin{7}; lambda_em = varargin{8};
        fcyl = varargin{9}; slitwidth = varargin{10};
        damping = varargin{11}; clipval = varargin{12}; stop_criterion = varargin{13};
        mem_percent = varargin{14}; gpu = varargin{15};

        %convert command line parameters from string to double
        if isdeployed
            dxy = str2double(strrep(dxy, ',', '.'));
            dz = str2double(strrep(dz, ',', '.'));
```

```
40              numit = str2double(strrep(numit, ',', '.'));
41              NA = str2double(strrep(NA, ',', '.'));
42              rf = str2double(strrep(rf, ',', '.'));
43              lambda_ex = str2double(strrep(lambda_ex, ',', '.'));
44              lambda_em = str2double(strrep(lambda_em, ',', '.'));
45              fcyl = str2double(strrep(fcyl, ',', '.'));
46              slitwidth = str2double(strrep(slitwidth, ',', '.'));
47              damping = str2double(strrep(damping, ',', '.'));
48              clipval = str2double(strrep(clipval, ',', '.'));
49              stop_criterion = str2double(strrep(stop_criterion, ',', '.'));
50              mem_percent = str2double(strrep(mem_percent, ',', '.'));
51              gpu = str2double(strrep(gpu, ',', '.'));
52          end
53
54          %start data processing
55
56          [CPUmem, GPUmem] = getmemory;
57          if gpu == 0
58              mem = CPUmem * mem_percent / 100;
59          else
60              mem = GPUmem * mem_percent / 100;
61          end
62
63          [nx, ny, nz] = getstackinfo(inpath);
64          if nx * ny * nz == 0
65              error('No valid TIFF files could be found!');
66          end
67
68          [tx, ty, tz] = autosplit(nx, ny, nz, mem);
69          if tx * ty * tz > 1
70              disp(['deconvolution split into ' num2str(tx) ' x ' num2str(ty) ' x ' num2str(tz) ' blocks.']);
71          end
72
73          process(inpath, tx, ty, tz, dxy, dz, numit, NA, rf, lambda_ex, lambda_em, fcyl, slitwidth, damping/100, clipval,
    stop_criterion, gpu);
74          if isdeployed
75              exit(0);
76          end
77      catch ME
```

```
78          %error handling
79          text = getReport(ME, 'extended', 'hyperlinks', 'off');
80          disp(text);
81          if isdeployed
82              exit(1);
83          end
84      end
85  end
86
87  function [CPU_mem, GPU_mem] = getmemory
88      [~, m] = memory;
89      CPU_mem = m.PhysicalMemory.Available;
90      try
91           check = gpuDevice; %check if CUDA is available
92           if (check.DeviceSupported == 1)
93              GPU_mem = check.TotalMemory;
94           else
95              GPU_mem = 0;
96           end
97      catch
98          GPU_mem = 0;
99      end
100 end
101
102 function showinfo()
103     disp('Usage: LsDeconv TIFDIR DELTAXY DELTAZ nITER NA RI LAMBDA_EX LAMBDA_EM FCYL SLITWIDTH DAMPING HISOCLIP STOP_CRIT
    MEM_PERCENT');
104     disp(' ');
105     disp('TIFFDIR: Directory containing the 2D-tiff files to be deconvolved(16-bit 0r 32bit float grayscale images
    supported).');
106     disp('The images are expected to be formated with numerical endings, as e.g. xx00001.tif, xx00002.tif, ...
    xx00010.tif....');
107     disp(' ');
108     disp('DELTAXY DELTAZ: xy- and z-Size of a voxel in nanometer. Choosing e.g. 250 500 means that a voxel is 250 nm x 250 nm
    wide in x- and y-direction');
109     disp('and 500 nm wide in z-direction (vertical direction). Values depend on the total magnification of the microscope and
    the camera chip.');
110     disp(' ');
111     disp('nITER: max. number of iterations for Lucy-Richardson algorithm. Deconvolution stops before if stop_crit is
```

```
reached.');
112     disp(' ');
113     disp('NA: numerical aperture of the objective.');
114     disp(' ');
115     disp('RI: refractive index of imaging medium and sample');
116     disp(' ');
117     disp('LAMBDA_EX: fluorescence excitation wavelength in nanometer.');
118     disp(' ');
119     disp('LAMBDA_EM: fluorescence emmision wavelength in nanometer.');
120     disp(' ');
121     disp('FCYL: focal length f in millimeter of the cylinder lens used for light sheet generation.');
122     disp(' ');
123     disp('SLITWIDTH: full width w in millimeter of the slit aperture placed in front of the cylinder lens. The NA of the
light sheet');
124     disp('generator system is calculated as NaLs = sin(arctan(w/(2*f))');
125     disp(' ');
126     disp('DAMPING: parameter between 0% and 10%. Increase value for images that are noisy. For images with');
127     disp('good signal to noise ratio the damping parameter should be set to zero (no damping at all)');
128     disp(' ');
129     disp('HISTOCLIP: percent value between 0 and 5 percent. If HISTOCLIP is set e.g. to 0.01% then the histogram of the
deconvolved');
130     disp('stack is clipped at the 0.01 and the 99.99 percentile and the remaininginte intensity values are scaled to the full
range (0...65535');
131     disp('in case of of 16 bit images, and 0...Imax in case of 32 bit float images, where Imax is the highest intensity
value');
132     disp('occuring in the source stack');
133     disp(' ');
134     disp('STOP_CRIT: I the pecentual change to the last iteration step becomes lower than STOP_CRIT the deconvolution of the
current');
135     disp('block is finished. If STOP_CRIT is e.g. set to 2% then the iteration stops, if there is less than 2% percent');
136     disp('change compared to the last iteration step.');
137     disp(' ');
138     disp('MEM_PERCENT: percent of RAM (or GPU memory, respectivbely, that can maximally by occopied by a data block. If the
size of the image stack');
139     disp('is larger than MEM_PERCENT * RAMSIZE / 100, the data set is split into blocks that are deconvolved sequentially and
then stitched.');
140     disp('A value of 4% usually is a good choice when working on CPU, a value of 50% when using the GPU. Decrease this value
if other memory consuming');
141     disp('programs are active.');
```

```
142         disp(' ');
143         disp('GPU: 0 = peform convolutions on CPU, 1 = perform convolutions on GPU');
144     end
145
146     %determine the required number of blocks that are deconvolved sequentially
147     function [tx, ty, tz] = autosplit(npix_x, npix_y, npix_z, maxblocksize)
148         tx = 0; ty = 0; tz = 0;
149         tz_max = max(floor(npix_z / 100), 1);
150
151         if maxblocksize == 0
152             return
153         end
154
155         while true
156             while true
157                 if npix_x / tx >= npix_y / ty
158                     tx = tx + 1;
159                 else
160                     ty = ty + 1;
161                 end
162
163                 if tx >= 99 || tz_max >= 99
164                     error('AutoTiles: error during splitting occured!');
165                 end
166
167                 for tz = 1 : tz_max
168                     bytes = ceil(npix_x / tx) * ceil(npix_y / ty) * ceil(npix_z / tz) * 4;
169                     if bytes <= maxblocksize
170                         return
171                     end
172                 end
173             end
174         end
175     end
176
177     function  process(inpath, tx, ty, tz, dxy, dz, numit, NA, rf, lambda_ex, lambda_em, fcyl, slitwidth, damping, ...
178                 clipval, stop_criterion, gpu)
179         tic;
180         %generate PSF
```

```
181         Rxy = 0.61 * lambda_em / NA;
182         dxy_corr = min(dxy, Rxy / 3);
183         [psf, nxy, nz, FWHMxy, FWHMz] = LsMakePSF(dxy_corr, dz, NA, rf, lambda_ex, lambda_em, fcyl, slitwidth);
184
185         %start deconvolution
186         scal  = deconvolve(inpath, psf, numit, damping, tx, ty, tz, clipval, stop_criterion, dxy, dz, gpu);
187
188         %write parameter info file
189         disp('generating info file...');
190         fid = fopen([inpath '\deconvolved\DECONV_parameters.txt'],'w');
191         fprintf(fid, '%s\r\n',['deconvolution finished at ' datestr(now)]);
192         fprintf(fid, '%s\r\n',['data processed on GPU: ' num2str(gpu)]);
193         fprintf(fid, '%s\r\n',['elapsed time: ' datestr(datenum(0,0,0,0,0, toc),'HH:MM:SS')]);
194         fprintf(fid, '%s\r\n', '');
195         fprintf(fid, '%s\r\n',['highest intensity value in deconvolved data: ' num2str(scal)]);
196         fprintf(fid, '%s\r\n',['focal length of cylinder lens (mm): ' num2str(fcyl)]);
197         fprintf(fid, '%s\r\n',['width of slit aperture (mm): ' num2str(slitwidth)]);
198         fprintf(fid,'%s\r\n', ['histogram clipping value (%): ' num2str(clipval)]);
199         fprintf(fid, '%s\r\n',['numerical aperture: ' num2str(NA)]);
200         fprintf(fid, '%s\r\n',['excitation wavelength (nm): ' num2str(lambda_ex)]);
201         fprintf(fid, '%s\r\n',['emission wavelength (nm): ' num2str(lambda_em)]);
202         fprintf(fid, '%s\r\n',['refractive index: ' num2str(rf)]);
203         fprintf(fid, '%s\r\n',['max. iterations: ' num2str(numit)]);
204         fprintf(fid, '%s\r\n',['damping factor (%): ' num2str(damping)]);
205         fprintf(fid, '%s\r\n',['stop criterion (%): ' num2str(stop_criterion)]);
206         fprintf(fid, '%s\r\n', '');
207         fprintf(fid, '%s\r\n',['source data folder: ' inpath]);
208         fprintf(fid, '%s\r\n',['number of blocks (x y z): ' num2str(tx)  ' x ' num2str(ty) ' x ' num2str(tz)]);
209         fprintf(fid, '%s\r\n', '');
210         fprintf(fid, '%s\r\n',['voxel size : ' num2str(dxy)  ' nm x ' num2str(dxy) ' nm x ' num2str(dz) ' nm']);
211         fprintf(fid, '%s\r\n',['size of PSF (pixel): ' num2str(nxy)  ' x ' num2str(nxy) ' x ' num2str(nz)]);
212         fprintf(fid, '%s\r\n',['FWHHM of PSF lateral (nm): ' num2str(FWHMxy)]);
213         fprintf(fid, '%s\r\n',['FWHHM of PSF axial (nm): ' num2str(FWHMz)]);
214         Rxy = 0.61 * lambda_em / NA;
215         Rz = (2 * lambda_em * rf) / NA^2;
216         fprintf(fid, '%s\r\n',['Rayleigh range of objective lateral (nm): ' num2str(Rxy)]);
217         fprintf(fid, '%s\r\n',['Rayleigh range of objective axial (nm): ' num2str(Rz)]);
218         fclose(fid);
219
```

```
220         disp(['deconvolution of: ' strrep(inpath, '\', '/') ' finished successfully']);
221         disp(['elapsed time: ' datestr(datenum(0,0,0,0,0, toc),'HH:MM:SS')]);
222         disp('------------------------------------------------------------------------------------------');
223     end
224
225     function scal = deconvolve(inpath, psf, numit, damping, numxblocks, numyblocks, numzblocks, clipval, stop_criterion, dxy, dz,
        gpu)
226         [info.x, info.y, info.z] = getstackinfo(inpath);
227         [p1, p2] = split(info, numxblocks, numyblocks, numzblocks);
228         %p1(blocknr, 1) : xstart; p1(blocknr, 2) : ystart; p1(blocknr, 3) : zstart
229         %p2(blocknr, 1) : xend; p2(blocknr, 2) : yend; p2(blocknr, 3) : zend
230
231         blocklist = strings(size(p1, 1), 1);
232         rawmax = 0; deconvmax = 0; deconvmin = Inf;
233         x = 1; y = 2; z = 3;
234
235         for i = 1 : size(blocklist, 1)
236             %begin processing next block
237
238             disp( ['loading block ' num2str(i) ' from ' num2str(size(blocklist, 1))]);
239
240             %load next block of data
241             startp = p1(i, :); endp = p2(i, :);
242             %startpos(x), startpos(y), startps(z) : start coordinates of currrent block (i)
243             %endpos(x), endpos(y), endpos(z) : end coordinates of current block
244
245             %load next blöock into memory
246             x1 = startp(x); x2 = endp(x);
247             y1 = startp(y); y2 = endp(y);
248             z1 = startp(z); z2 = endp(z);
249             bl = load_block(inpath, x1, x2, y1, y2, z1, z2);
250
251             %get min-max of raw data stack
252             temp = max(bl(:));
253             if temp > rawmax
254                 rawmax = temp;
255             end
256
257             %deconvolve current block of data
```

```
258             disp(['processing block ' num2str(i) ' from ' num2str(size(blocklist, 1))]);
259             bl = process_block(bl, psf, numit, damping, stop_criterion, gpu);
260 
261             %find maximum value in processed data
262             temp = max(bl(:));
263             if temp > deconvmax
264                 deconvmax = temp;
265             end
266             temp = min(bl(:));
267             if temp < deconvmin
268                 deconvmin = temp;
269             end
270 
271             if numxblocks * numyblocks * numzblocks > 1 % there is more than one block
272                 disp(['saving block ' num2str(i) ' from ' num2str(size(blocklist, 1))]);
273 
274                 %save block to disk
275                 blocklist(i) = [tempdir 'bl' num2str(i) '_temp.mat'];
276                 save(blocklist(i), 'bl', '-v7.3', '-nocompression');
277             end
278         end
279 
280         if clipval > 0
281             %estimate the global histogram and upper and lower clipping values
282             nbins = 1e6;
283             binwidth = deconvmax / nbins;
284             bins = 0 : binwidth : deconvmax;
285 
286             %calculate cumulative histogram by scanning all blocks
287             disp('calculating histogram...');
288 
289             chist = cumsum(histcounts(bl, bins)); %histogram from last block
290             if numxblocks * numyblocks * numzblocks > 1
291                 for i = size(blocklist, 1) - 1 : -1 : 1
292                     S = load(blocklist(i), 'bl');
293                     chist = chist + cumsum(histcounts(S.bl, bins));
294                 end
295                 clear S;
296             end
```

```
297
298         %determine upper and lower histogram clipping values
299         chist = chist / max(chist) * 100; %normalize cumulative histogram to 0..100%
300
301         low_clip = findClosest(chist, clipval) * binwidth;
302         high_clip = findClosest(chist, 100-clipval) * binwidth;
303     end
304
305     %make folder for results and write tiff-images
306     outpath = [inpath '\deconvolved'];
307     if exist(outpath, 'dir') == 0
308         mkdir(outpath);
309     else
310         delete([outpath '\*.tif']);
311     end
312
313     %mount data and save data layeer by layer
314     blnr = 1; imagenr = 1;
315     for i = 1 : numzblocks
316         disp(['mounting layer ' num2str(i) ' from ' num2str(numzblocks)]);
317
318         %load and mount next layer of images
319         if numxblocks * numyblocks * numzblocks > 1
320             R = zeros(info.x, info.y, p2(blnr, z)- p1(blnr, z)+1, 'single');
321             for j = 1 : numxblocks * numyblocks
322                 S = load(blocklist(blnr), 'bl');
323                 delete(convertStringsToChars(blocklist(blnr)));
324
325                 R( p1(blnr, x) : p2(blnr, x), p1(blnr, y) : p2(blnr, y), :) = S.bl;
326                 blnr = blnr + 1;
327             end
328             clear S;
329         else %if there is only one block of data
330             R = bl;
331             clear bl;
332         end
333
334         %rescale deconvolved data
335         if rawmax <= 65535
```

```
336             scal = 65535;
337         else
338             scal = rawmax; %scale to maximum of input data
339         end
340
341         if clipval > 0
342             %perform histogram clipping
343             R(R < low_clip) = low_clip;
344             R(R > high_clip) = high_clip;
345             R = (R - low_clip) ./ (high_clip - low_clip) .* scal;
346         else
347             %otherwise scale to min..max
348             R = (R - deconvmin) ./ (deconvmax - deconvmin) * scal;
349         end
350
351         %write images to folder
352         disp('saving images...');
353
354         for k = 1 : size(R, 3)
355             s = num2str(imagenr);
356             while length(s) < 6
357                 s = strcat('0', s);
358             end
359             fullname = [outpath '\deconv_' s '.tif'];
360
361             if rawmax <= 65535 %16bit data
362                 im = uint16(squeeze(R(:, :, k)));
363                 imwrite(im', fullname);
364             else %32 bit data
365                 im = squeeze(R(:, :, k));
366                 writeTiff32(im', fullname) %R must be single;
367             end
368             imagenr = imagenr + 1;
369         end
370     end
371 end
372
373 function bl = load_block(inpath, start_x, end_x, start_y, end_y, start_z, end_z)
374     filelist = dir(fullfile(inpath, '\*.tif'));
```

```
375
376         nx = end_x - start_x;
377         ny = end_y - start_y;
378         nz = end_z - start_z;
379
380         bl = zeros(nx+1, ny+1, nz+1, 'single');
381         for k = 1 : nz+1
382             im = im2single((imread([inpath '\' filelist((k-1)+start_z).name],
    'PixelRegion',{[start_y,end_y],[start_x,end_x]})));
383             bl(:, :, k) = im';
384         end
385     end
386
387     function block = process_block(block, psf, numit, damping, stopcrit, gpu)
388         %for efficiency of FFT pad data in a way that the largest prime factor becomes <= 5
389         blx = size(block, 1); bly = size(block, 2); blz = size(block, 3);
390         pad_x = 0.5 * (findGoodFFTLength(blx + 4 * size(psf, 1)) - blx);
391         pad_y = 0.5 * (findGoodFFTLength(bly + 4 * size(psf, 2)) - bly);
392         pad_z = 0.5 * (findGoodFFTLength(blz + 4 * size(psf, 3)) - blz);
393
394         block = padarray(block, [floor(pad_x) floor(pad_y) floor(pad_z)], 'pre', 'symmetric');
395         block = padarray(block, [ceil(pad_x) ceil(pad_y) ceil(pad_z)], 'post', 'symmetric');
396
397         %deconvolve block using Lucy-Richardson algorithm
398         if gpu
399             block = deconGPU(block, psf, numit, damping, stopcrit);
400         else
401             block = deconCPU(block, psf, numit, damping, stopcrit);
402         end
403
404         %remove padding
405         block = block(floor(pad_x) : end-ceil(pad_x)-1, floor(pad_y) : end-ceil(pad_y)-1, floor(pad_z) : end-ceil(pad_z)-1);
406     end
407
408     %provides coordinates of subblocks after splitting
409     function [p1, p2] = split(info, nx, ny, nz)
410         xw = ceil(info.x / nx);
411         yw = ceil(info.y / ny);
412         zw = ceil(info.z / nz);
```

```
413
414         p1 = zeros(nx*ny*nz, 3);
415         p2 = zeros(nx*ny*nz, 3);
416
417         n = 0;
418         for i = 0 : nz-1
419             zs = i * zw + 1;
420             for j = 0 : ny-1
421                 ys = j * yw + 1;
422                 for k = 0 : nx-1
423                     xs = k * xw + 1;
424                     n = n + 1;
425                     p1(n, 1) = xs;
426                     p2(n, 1) = min([xs + xw - 1, info.x]);
427
428                     p1(n, 2) = ys;
429                     p2(n, 2) = min([ys + yw - 1, info.y]);
430
431                     p1(n, 3) = zs;
432                     p2(n, 3) = min([zs + zw - 1, info.z]);
433                 end
434             end
435         end
436     end
437
438     %writes 32bit float tiff-imges
439     function writeTiff32(img, fname)
440         t = Tiff(fname, 'w');
441         tag.ImageLength = size(img, 1);
442         tag.ImageWidth = size(img, 2);
443         tag.Compression = Tiff.Compression.LZW;
444         tag.SampleFormat = Tiff.SampleFormat.IEEEFP;
445         tag.Photometric = Tiff.Photometric.MinIsBlack;
446         tag.BitsPerSample = 32;
447         tag.SamplesPerPixel = 1;
448         tag.PlanarConfiguration = Tiff.PlanarConfiguration.Chunky;
449         t.setTag(tag);
450         t.write(img);
451         t.close();
```

```
452 end
453
454 function index = findClosest(data, x)
455     [~,index] = min(abs(data-x));
456 end
457
458 function x = findGoodFFTLength(x)
459     while max(factor(x)) > 5
460         x = x + 1;
461     end
462 end
463
464 %Lucy-Richardson deconvolution
465 function deconvolved = deconCPU(stack, psf, niter, lambda, stop_criterion)
466     deconvolved = stack;
467     OTF = single(psf2otf(psf, size(stack)));
468
469     R = 1/26 * ones(3, 3, 3, 'single'); R(2,2,2) = single(0);
470
471     for i = 1 : niter
472         denom = convFFT(deconvolved, OTF);
473         denom(denom < eps('single')) = eps('single'); % protect against division by zero
474
475         if lambda == 0
476             deconvolved_new = convFFT(stack ./ denom, conj(OTF)) .* deconvolved;
477         else
478             deconvolved_new = (1 - lambda) .* convFFT(stack ./ denom, conj(OTF)) .* deconvolved ...
479                 + lambda .* convn(deconvolved, R, 'same');
480         end
481
482         %estimate quality criterion
483         delta = sqrt(sum((deconvolved(:) - deconvolved_new(:)).^2));
484         if i == 1
485             delta_rel = 0;
486         else
487             delta_rel = (deltaL - delta) / deltaL * 100;
488         end
489
490         deconvolved = deconvolved_new;
```

```
491             deltaL = delta;
492 
493             disp(['iteration: ' num2str(i), ' delta: ' num2str(delta_rel, 3)]);
494 
495             if i > 1 && delta_rel <= stop_criterion
496                 disp('stop criterion reached. Finishing iterations.');
497                 break
498             end
499         end
500 
501         %get rid of imaginary artifacts
502         deconvolved = abs(deconvolved);
503     end
504 
505     function deconvolved = deconGPU(stack, psf, niter, lambda, stop_criterion)
506         deconvolved = stack;
507         psf_inv = psf(end:-1:1,end:-1:1, end:-1:1); % spatially reversed psf
508 
509         R = 1/26 * ones(3, 3, 3, 'single'); R(2,2,2) = single(0);
510 
511         for i = 1 : niter
512             denom = convGPU(deconvolved, psf);
513             denom(denom < eps('single')) = eps('single'); % protect against division by zero
514 
515             if lambda == 0
516                 deconvolved_new = convGPU(stack ./ denom, psf_inv) .* deconvolved;
517             else
518                 deconvolved_new = (1 - lambda) .* convGPU(stack ./ denom, psf_inv) .* deconvolved ...
519                     + lambda .* convn(deconvolved, R, 'same');
520             end
521 
522             %estimate quality criterion
523             delta = sqrt(sum((deconvolved(:) - deconvolved_new(:)).^2));
524             if i == 1
525                 delta_rel = 0;
526             else
527                 delta_rel = (deltaL - delta) / deltaL * 100;
528             end
529 
```

```
530             deconvolved = deconvolved_new;
531             deltaL = delta;
532 
533             disp(['iteration: ' num2str(i), ' delta: ' num2str(delta_rel, 3)]);
534 
535             if i > 1 && delta_rel <= stop_criterion
536                 disp('stop criterion reached. Finishing iterations.');
537                 break
538             end
539         end
540 
541         %get rid of imaginary artifacts
542         deconvolved = abs(deconvolved);
543     end
544 
545     %deconvolve with OTF
546     function R = convFFT(data , otf)
547         R = ifftn(otf .* fftn(data));
548     end
549 
550     function R = convGPU(data, psf)
551         R = gather(convn(gpuArray(data), psf, 'same'));
552     end
553 
554     %calculates a theoretical point spread function
555     function [psf, nxy, nz, FWHMxy, FWHMz] = LsMakePSF(dxy, dz, NA, nf, lambda_ex, lambda_em, fcyl,slitwidth)
556         disp('calculating PSF...');
557         [nxy, nz, FWHMxy, FWHMz] = DeterminePSFsize(dxy, dz, NA, nf, lambda_ex, lambda_em, fcyl, slitwidth);
558 
559         %construct psf
560         NAls = sin(atan(slitwidth / (2 * fcyl)));
561         psf = samplePSF(dxy, dz, nxy, nz, NA, nf, lambda_ex, lambda_em, NAls);
562         disp('ok');
563     end
564 
565 
566     %determine the required grid size (xyz) for psf sampling
567     function [nxy, nz, FWHMxy, FWHMz] = DeterminePSFsize(dxy, dz, NA, nf, lambda_ex, lambda_em, fcyl, slitwidth)
568         %Size of PSF grid is gridsize (xy z) times FWHM
```

```
569         gridsizeXY = 2;
570         gridsizeZ = 2;
571 
572         NAls = sin(atan(0.5 * slitwidth / fcyl));
573         halfmax = 0.5 .* LsPSFeq(0, 0, 0, NA, nf, lambda_ex, lambda_em, NAls);
574 
575         %find zero crossings
576         fxy = @(x)LsPSFeq(x, 0, 0, NA, nf, lambda_ex, lambda_em, NAls) - halfmax;
577         fz = @(x)LsPSFeq(0, 0, x, NA, nf, lambda_ex, lambda_em, NAls) - halfmax;
578         FWHMxy = 2 * abs(fzero(fxy, 100));
579         FWHMz = 2 * abs(fzero(fz, 100));
580 
581         Rxy = 0.61 * lambda_em / NA;
582         dxy_corr = min(dxy, Rxy / 3);
583 
584         nxy = ceil(gridsizeXY * FWHMxy / dxy_corr);
585         nz = ceil(gridsizeZ * FWHMz / dz);
586 
587         %ensure that the grid dimensions are odd
588         if mod(nxy, 2) == 0
589             nxy = nxy + 1;
590         end
591         if mod(nz, 2) == 0
592             nz = nz + 1;
593         end
594     end
595 
596     function psf = samplePSF(dxy, dz, nxy, nz, NA_obj, rf, lambda_ex, lambda_em, NA_ls)
597         if mod(nxy, 2) == 0 || mod(nz, 2) == 0
598             error('function samplePSF: nxy and nz must be odd!');
599         end
600 
601         psf = zeros((nxy - 1) / 2 + 1, (nxy - 1) / 2 + 1, (nz - 1) / 2 + 1, 'single');
602         for z = 0 : (nz - 1) / 2
603             for y = 0 : (nxy - 1) / 2
604                 for x = 0 : (nxy - 1) / 2
605                    psf(x+1, y+1, z+1) = LsPSFeq(x*dxy, y*dxy, z*dz, NA_obj, rf, lambda_ex, lambda_em, NA_ls);
606                 end
607             end
```

```
608         end
609
610         %Since the PSF is symmetrical around all axes only the first Octand
611         %is calculated for computation efficiency. The other 7 Octands are obtained by mirroring around
612         %the respecitve axes
613         psf = mirror8(psf);
614
615         %normalize psf to integral one
616         psf = psf ./ sum(psf(:));
617     end
618
619     function R = mirror8(p1)
620         %mirrors the content of the first quadrant to all other quadrants to
621         %obtain the complete PSF.
622
623         sx = 2 * size(p1, 1) - 1; sy = 2 * size(p1, 2) - 1; sz = 2 * size(p1, 3) - 1;
624         cx = ceil(sx / 2); cy = ceil(sy / 2); cz = ceil(sz / 2);
625
626         R = zeros(sx, sy, sz, 'single');
627         R(cx:sx, cy:sy, cz:sz) = p1;
628         R(cx:sx, 1:cy, cz:sz) = flip3D(p1, 0, 1 ,0);
629         R(1:cx, 1:cy, cz:sz) = flip3D(p1, 1, 1, 0);
630         R(1:cx, cy:sy, cz:sz) = flip3D(p1, 1, 0, 0);
631         R(cx:sx, cy:sy, 1:cz) = flip3D(p1, 0, 0, 1);
632         R(cx:sx, 1:cy, 1:cz) =  flip3D(p1, 0, 1 ,1);
633         R(1:cx, 1:cy, 1:cz) =  flip3D(p1, 1, 1, 1);
634         R(1:cx, cy:sy, 1:cz) =  flip3D(p1, 1, 0, 1);
635     end
636
637     %utility function for mirror8
638     function R = flip3D(data, x, y, z)
639         R = data;
640         if x
641             R = flip(R, 1);
642         end
643         if y
644             R = flip(R, 2);
645         end
646         if z
```

```
647             R = flip(R, 3);
648         end
649     end
650
651     %calculates PSF at point (x,y,z)
652     function R = LsPSFeq(x, y, z, NAobj, n, lambda_ex, lambda_em, NAls)
653         R = PSF(z, 0, x, NAls, n, lambda_ex) .* PSF(x, y, z, NAobj, n, lambda_em);
654     end
655
656     %utility function for LsPSFeq
657     function R = PSF(x, y, z, NA, n, lambda)
658         f2 = @(p)f1(p, x, y, z, lambda, NA, n);
659         R = 4 .* abs(integral(f2, 0, 1, 'AbsTol',1e-3)).^2;
660     end
661
662     %utility function for LsPSFeq
663     function R = f1(p, x, y, z, lambda, NA, n)
664         R = besselj(0, 2 .* pi .* NA .* sqrt(x.^2 + y.^2) .* p ./ (lambda .* n))...
665             .* exp(1i .* (-pi .* p.^2 .* z .* NA.^2) ./ (lambda .* n.^2)) .* p;
666     end
667
668     function [x, y, z] = getstackinfo(datadir)
669         filelist = dir(fullfile(datadir, '\*.tif'));
670         if numel(filelist) == 0
671             x = 0; y = 0; z = 0;
672         else
673             test = (imread([datadir '\' filelist(1).name]))';
674             x = size(test, 1);
675             y = size(test, 2);
676             z = numel(filelist);
677         end
678     end
679
680
681
682
683
684
685
```
